# Macrophage Colony Stimulating Factor Derived from CD4^+^ T Cells Contributes to Control of a Blood-Borne Infection

**DOI:** 10.1371/journal.ppat.1006046

**Published:** 2016-12-06

**Authors:** Mary F. Fontana, Gabrielly L. de Melo, Chioma Anidi, Rebecca Hamburger, Chris Y. Kim, So Youn Lee, Jennifer Pham, Charles C. Kim

**Affiliations:** Division of Experimental Medicine, Department of Medicine, University of California, San Francisco, San Francisco, CA, United States of America; Francis Crick Institute, UNITED KINGDOM

## Abstract

Dynamic regulation of leukocyte population size and activation state is crucial for an effective immune response. In malaria, *Plasmodium* parasites elicit robust host expansion of macrophages and monocytes, but the underlying mechanisms remain unclear. Here we show that myeloid expansion during *P*. *chabaudi* infection is dependent upon both CD4^+^ T cells and the cytokine Macrophage Colony Stimulating Factor (MCSF). Single-cell RNA-Seq analysis on antigen-experienced T cells revealed robust expression of *Csf1*, the gene encoding MCSF, in a sub-population of CD4^+^ T cells with distinct transcriptional and surface phenotypes. Selective deletion of *Csf1* in CD4^+^ cells during *P*. *chabaudi* infection diminished proliferation and activation of certain myeloid subsets, most notably lymph node-resident CD169^+^ macrophages, and resulted in increased parasite burden and impaired recovery of infected mice. Depletion of CD169^+^ macrophages during infection also led to increased parasitemia and significant host mortality, confirming a previously unappreciated role for these cells in control of *P*. *chabaudi*. This work establishes the CD4^+^ T cell as a physiologically relevant source of MCSF *in vivo;* probes the complexity of the CD4^+^ T cell response during type 1 infection; and delineates a novel mechanism by which T helper cells regulate myeloid cells to limit growth of a blood-borne intracellular pathogen.

## Author Summary

Malaria, caused by *Plasmodium* parasites, places a huge disease burden on humankind. Efforts to develop an effective vaccine for this pathogen are hampered by a poor understanding of the kinds of immune responses needed for protection. When infected with *Plasmodium*, humans and other mammalian hosts develop greatly increased numbers of certain white blood cells called myeloid cells, but the extent to which this expansion is important for fighting infection is unknown, as are the factors that regulate it. In this work, we show that expansion of myeloid cells during malaria is critical for control of parasite replication and host recovery. Myeloid expansion and control of infection require production of the signaling protein Macrophage Colony Stimulating Factor (MCSF) by multiple sources, including CD4^+^ T cells, which had not previously been known to make this protein in a physiological setting. This study reveals a new mechanism through which immune cells cooperate to control malaria, and may guide future attempts to generate protective immune responses against this devastating pathogen.

## Introduction

During infection, specific immune subsets must proliferate robustly to generate a population of effector cells large enough to contain the microbial threat. A well-characterized example is the dramatic expansion of antigen-specific T lymphocytes, whose numbers may increase over a thousand-fold during the course of an immune response [1]. Myeloid cells also undergo expansion in a number of infections [2–7]. The trafficking and recruitment of monocytes into infected tissues have been defined in detail [8]; however, the mechanisms controlling myeloid proliferation during infection are much less well understood. Under homeostatic conditions the survival and renewal of the mononuclear phagocyte lineage, which includes monocytes and macrophages, is controlled primarily by the cytokine Macrophage Colony Stimulating Factor (MCSF) [9,10]. In addition, MCSF can activate myeloid cells *in vitro* [9]. But the extent to which MCSF also regulates macrophage and monocyte proliferation and activation under inflammatory conditions is not clearly established, in part because the grave baseline defects of mice genetically deficient in this cytokine have complicated such analysis [11].

Infection with protozoan parasites of the genus *Plasmodium* results in a dramatic expansion of monocytes and macrophages that has long been considered a hallmark of malaria disease in humans and other mammalian hosts [12–15]. In mouse models employing rodent-adapted parasites, myeloid expansion has been shown to involve IL-27-dependent proliferation of hematopoietic stem cells in the bone marrow [16] and interferon gamma (IFN-γ)-dependent mobilization of multipotent myeloid progenitor cells into the spleen [5,17], where they can give rise to monocytes and, presumably, macrophages. However, the cells and cytokines that regulate differentiation and proliferation downstream of these early progenitor stages remain undefined. Recent work has demonstrated that tissue-resident macrophages can proliferate *in situ* during helminth infection through a process requiring the type 2 cytokine interleukin-4 (IL-4) [6,7]. These findings raise the question of whether macrophages and monocytes undergo local expansion in response to type 1 pathogens such as *Plasmodium*, and if so, what factors regulate this process.

In this work, we investigate the causes and consequences of myeloid proliferation and activation during infection with *P*. *chabaudi*. We find that MCSF derived from multiple sources drives proliferation of macrophages and monocytes in infected mice; moreover, the full expansion and activation of certain subsets specifically requires MCSF derived from circulating CD4^+^ T cells, which have not previously been demonstrated to produce this cytokine in a physiological context. We measure the inducible upregulation of *Csf1* in antigen-experienced CD4^+^ T cells from infected mice, and show that CD4^+^ T cell-derived MCSF is important for control of parasitemia and recovery of host health late in infection, coinciding with the kinetics of maximal myeloid expansion. Finally, we demonstrate a previously unappreciated role for CD169^+^ macrophages, which are diminished in mice lacking MCSF production in CD4^+^ T cells, in restriction of *P*. *chabaudi* parasite burden and host survival. Thus, this study establishes a new physiological source of MCSF *in vivo*, and delineates a novel mechanism by which CD4^+^ T cells regulate the myeloid compartment to control a blood-borne intracellular infection.

## Results

### Macrophages remain critical for control of *Plasmodium* parasitemia during the resolution phase of infection

**Fig 1:**
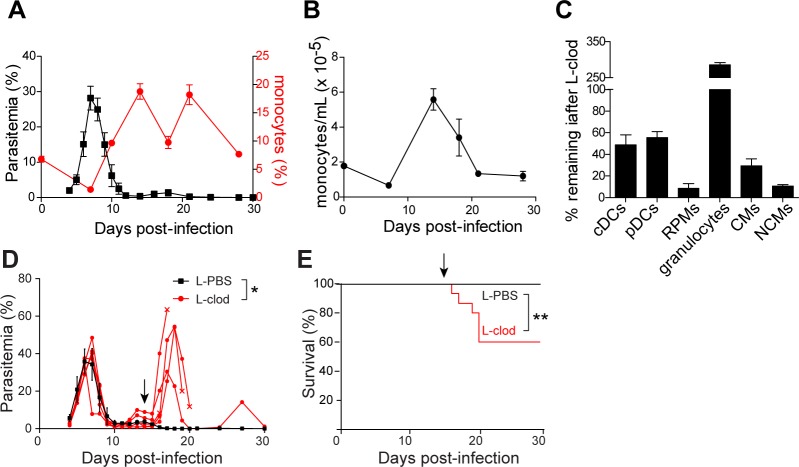
An expanded myeloid compartment restricts *Plasmodium* parasitemia during the resolution phase of infection. (A) Parasitemia (% of RBCs infected; black line) and monocyte frequencies (red line) were monitored in the blood of wild-type mice following infection with *P*. *chabaudi*. Mean +/- SEM of 4 mice is depicted. (B) Absolute numbers of blood monocytes (classical and nonclassical combined) in infected mice (mean +/- SEM; n = 4). (C-E) Infected mice were treated with liposome-encapsulated clodronate (L-clod) or control liposomes (L-PBS) 14 d post-infection (d.p.i.) with blood-stage *P*. *chabaudi*. (C) Depletion of the indicated myeloid populations was assessed in the spleen 24 h after L-clod treatment. Graph depicts the percentage of each subset remaining in L-clod-treated mice relative to L-PBS-treated mice. (D) Parasitemia and (E) survival were monitored. In (D), red lines depict individual L-clod-treated mice, with each x indicating mortality; black line represents mean parasitemia of 5 control mice. Black arrows indicate time of liposome treatment. In (D), data depicted are representative of two biological replicates; *, p < 0.05 by Mann-Whitney test. In (E), data are pooled from two biological replicates (n = 10 mice per group); ***, p < 0.001 by Mantel-Cox test.

In the *P*. *chabaudi* blood-stage model of malaria, parasitemia peaks approximately 7 days post-infection (d.p.i.), after which it is rapidly controlled to low levels (<5% of red blood cells infected) (**Fig 1A,** black line). For this study, we divided the infection conceptually into two phases: the acute phase, during which parasitemia peaks, and the resolution phase, from approximately 10–30 d.p.i., after acute parasitemia has been controlled but before the infection has been cleared to subpatent levels. It has long been observed that myeloid cells expand in number and frequency during the blood stage of *Plasmodium* infection [3,12–14], and previous studies demonstrate that phagocytic cells, presumed to include macrophages, are involved in control of parasitemia during the acute phase of infection [18,19]. However, in the *P*. *chabaudi* model, myeloid expansion does not reach its peak until the resolution phase, i.e. approximately two weeks post-infection, well after acute parasitemia has been controlled [3,5] (**Fig 1A,** red line, and **1B**). Additionally, macrophage surface activation markers remain elevated for days after control of acute infection [20]. Therefore, we considered the hypothesis that macrophages might also be important for limiting parasitemia during the resolution phase. To test whether this is the case, we depleted phagocytic cells in *P*. *chabaudi*-infected mice 14 d.p.i. A small recrudescence typically occurs around this time (**Fig 1A**). We first examined the efficiency of depletion for a number of myeloid subsets in the blood and spleen, including classical monocytes (CMs), defined by high expression of Ly6C; nonclassical (Ly6C^lo^) monocytes (NCMs); and red pulp macrophages (RPMs), the most abundant population of splenic macrophages [21] (gating strategy, **S1 Fig**). Flow cytometric analysis of myeloid populations in the spleen and blood following treatment revealed efficient depletion of RPMs and monocytes, whereas conventional and plasmacytoid dendritic cells were partially depleted and granulocytes actually expanded (**Fig 1C**). We did not observe alterations in non-myeloid cell frequencies in treated mice. Strikingly, mice depleted of phagocytes late in infection experienced a rapid resurgence of parasitemia, rising as high as 60% (**Fig 1D**), accompanied by significant mortality (**Fig 1E**). These results demonstrate that phagocytic cells, most likely macrophages and/or monocytes, remain critical for control of parasitemia during the resolution phase of infection, coinciding with the kinetics of their maximum expansion and sustained activation.

Depletion of myeloid cells could affect parasitemia directly, e.g. through loss of phagocytic and microbicidal capacity, or indirectly through effects on adaptive cells such as T cells. To search for possible effects on T cell activation, we performed intracellular cytokine staining for IFN-γ in CD4^+^ T cells following late phagocyte depletion in infected mice. However, we could not detect IFN-γ protein in T cells from either control or myeloid-depleted mice at this late timepoint, consistent with previous reports showing that cytokine production peaks approximately 6 d.p.i. and is virtually undetectable by two weeks [22–24]. It is possible that late depletion of myeloid cells results in defects in T cell functions other than production of IFN-γ and IL-2; however, we note that dendritic cell activation, antigen presentation, and activation of adaptive responses occur as early as 5–7 d.p.i. in the mouse model [24–26], and robust B and T cell responses are well-established by 14 d.p.i. [27–29]. Taken together, these findings make it less likely that the observed effects on parasite control are due to impacts on antigen-presenting cells and disruption of adaptive responses. Instead, we favor the hypothesis that late phagocyte depletion crucially targets macrophages and/or monocytes, which are required during the resolution phase to phagocytose and clear infected red blood cells.

### CD4^+^ T cells inducibly express *Csf1* during *P*. *chabaudi* infection

**Fig 2:**
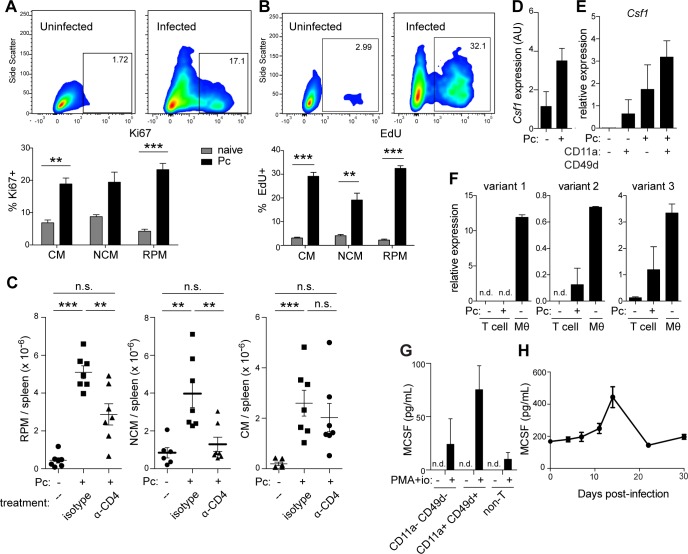
CD4^+^ T cells inducibly express *Csf1* and promote expansion of myeloid cells during infection with *P*. *chabaudi*. (A) Ki67 and (B) EdU labeling in splenic myeloid cells from naive wild-type mice or mice infected for 14 d with *P*. *chabaudi*. Representative plots from one uninfected and one infected mouse are shown, gated on RPM. Graphs show mean +/- SEM of 3 mice per group. **, p < 0.01; ***, p < 0.001 by *t*-test. (C) Wild-type mice were treated with α-CD4 or an isotype control antibody 4 d.p.i. Spleens were harvested 14 d.p.i., and myeloid populations were enumerated by flow cytometry. Dots depict individual mice pooled from two independent biological replicates; bars indicate mean and SEM. Statistical significance was determined by one-way ANOVA with Tukey’s post-test. n.s., not significant; **, p < 0.01; ***, p < 0.001. (D) *Csf1* transcript levels were measured by microarray in bulk CD4^+^ T cells from naive mice and in antigen-experienced (CD11a^+^ CD49d^+^) CD4^*+*^ T cells isolated from mice infected for 6 d with *P*. *chabaudi*. Mean + SD of four technical replicates is shown. (E) *Csf1* transcript was assessed by RT-qPCR in purified antigen-naive (CD11a^-^ CD49d^-^) and antigen-experienced CD4^+^ T cells from naive or infected (6 d.p.i.) mice. (F) Levels of individual *Csf1* transcript variants were measured by RT-qPCR in naive CD4^+^ T cells or antigen-experienced CD4^+^ T cells from infected mice 6 d.p.i. Bone marrow-derived macrophages (Mθ) treated with LPS (10 ng/mL, 4h) were used as a positive control for *Csf1* expression. (G) CD4^+^ T cells, sorted by CD11a and CD49d expression from infected mice 6 d.p.i., were restimulated with PMA and ionomycin (io) *in vitro*. MCSF was measured in supernatant by ELISA. TCRβ^-^ cells (non-T) were analyzed as a control. Mean + SEM from three technical replicates is shown; results are representative of two independent biological experiments. (H) MCSF was quantified in plasma from infected mice by ELISA. Mean +/- SEM is shown (n = 4). n.d., not detected. Pc, *P*. *chabaudi*-infected. RPM, red pulp macrophages. NCM, nonclassical monocytes. CM, classical monocytes.

The observed increase in macrophage and monocyte numbers in infected mice is due at least in part to proliferation and recruitment of progenitor cells from the bone marrow [16,17], but it also might involve local proliferation of monocytes and/or macrophages in the tissues, as has been documented in helminth infection [6]. To test whether differentiated myeloid cells proliferate locally during *P*. *chabaudi* infection, we assessed levels of the nuclear proliferation marker Ki67 in myeloid subsets from infected spleens. Whereas splenic RPMs, CMs, and NCMs from naïve mice largely lacked this marker, we detected significant Ki67 expression in all three subsets 14 d.p.i. with *P*. *chabaudi* (**Fig 2A**). Similarly, significant fractions of RPMs, CMs and NCMs from infected, but not naïve, mice incorporated the injected thymidine analog 5-ethynyl-2’-deoxyuridine (EdU), indicating active DNA synthesis (**Fig 2B**). Thus, myeloid expansion during malaria infection involves local proliferation of differentiated, tissue-resident cells.

We next investigated factors that might influence this proliferation of monocytes and macrophages during *P*. *chabaudi* infection. Among other subsets, we considered a role for CD4^+^ T helper cells, whose importance in controlling blood-stage *Plasmodium* has been suggested in humans [30–32] and demonstrated in mice [33–37]; infected mice depleted of CD4^+^ T cells exhibit defects in parasite control reminiscent of those observed after late macrophage depletion [35,28,37,38] (**Fig 1D**). Accordingly, we found that depletion of CD4^+^ T cells 4 d.p.i. disrupted the expansion of splenic RPMs and NCMs in infected mice, although CMs were unaffected (**Fig 2C**). The effect of CD4^+^ T cell depletion was not due to increased apoptosis of myeloid cells, since the frequency of apoptotic monocytes and macrophages was not increased in splenocytes from T cell-depleted mice (**S2 Fig**). We proceeded to examine mechanisms by which CD4^+^ T cells might drive this myeloid expansion.

Much research on the role of CD4^+^ T cells in malaria has focused on their abundant production of IFN-γ[23,39,24], and indeed, infected *Ifng* ^*-/-*^ mice have fewer splenic macrophages than wild-type mice during the acute phase of infection [39]. Nevertheless, several observations led us to consider the hypothesis that T cells might regulate myeloid cells through multiple mechanisms, in addition to production of IFN-γ. First, it was not clear to us how IFN-γ might promote proliferation of differentiated macrophages and monocytes; in fact, IFN-γ is generally considered to be anti-proliferative for these cell types [40], although it may stimulate proliferation of hematopoietic stem cells [5,16,41]. Second, one study using the related parasite *P*. *yoelii* found that protective macrophage populations were intact in infected mice lacking IFN-γ [19], suggesting the presence of additional mechanisms governing their activity.

Thus, to identify additional T cell-dependent factors that might regulate myeloid cells during infection, we performed transcriptome analysis on activated CD4^+^ T cells sorted from infected mice 6 d.p.i., when production of many cytokines peaks [22–24]. Lacking tools to detect antigen-specific cells, we used the integrins CD11a and CD49d as proxy markers for antigen-experienced cells, an approach that has been validated in this and other animal models [42,43] (gating strategy, **S3A Fig**). Unexpectedly, we detected significant expression of the gene *Csf1*, encoding MCSF, in antigen-experienced (CD11a^+^ CD49d^+^) CD4^+^ T cells from infected mice, but not bulk CD4^+^ T cells from naive mice (**Fig 2D**). Using quantitative RT-PCR, we confirmed that *Csf1* was upregulated in antigen-experienced CD4^+^ cells from infected mice, but not in antigen-naive cells from infected mice or in any CD4^+^ T cells from naive mice, regardless of antigen exposure (**Fig 2E**). The predominant transcript expressed was variant 3, which encodes a soluble form of the protein that can be secreted either as a glycoprotein or a proteoglycan, depending on differential proteolysis during export [10]. Smaller amounts of variant 2, encoding a membrane-bound form of the cytokine, were also detected (**Fig 2F**). Intracellular cytokine staining for MCSF has not been reported to our knowledge, and our attempts to measure MCSF protein in CD4^+^ T cells by flow cytometry were not successful. However, by performing ELISA on supernatants from antigen-experienced CD4^+^ T cells sorted from infected mice 6 d.p.i. and restimulated in culture, we confirmed the expression of MCSF protein in this population (**Fig 2G**). Simultaneously sorted TCRβ^-^ cells cultured in parallel did not produce significant amounts of MCSF, making it unlikely that MCSF production in sorted CD4^+^ T cell populations actually comes from contaminating myeloid cells (**Fig 2G**). In contrast to IFN-γ, which peaks around 6 d.p.i. and is virtually undetectable after two weeks [22–24], MCSF levels continued to increase in the plasma of infected mice for two weeks post-infection (**Fig 2H**), a time frame coincident with the peak of myeloid expansion (**Fig 1B**), consistent with the hypothesis that this cytokine might support the observed proliferation of myeloid cells at later timepoints.

### MCSF is expressed in a subset of activated CD4^+^ T cells that also express Th1 markers

During homeostasis, MCSF is predominantly produced by endothelial and stromal cells; under inflammatory conditions, it can also be made by activated monocytes and macrophages themselves [9,10]. Several early publications described *Csf1* expression in cultured CD4^+^ T cells stimulated extensively *in vitro* [44–47]. In addition, one study has reported detection of lymphocyte-associated MCSF in human lymph node tumor biopsies [48], and two others found evidence for *Csf1* production in decidual T cells during pregnancy [49,50]. Despite these examples, T cells are not generally considered to be a biologically relevant source of MCSF. Studies of baseline expression in an MCSF reporter mouse did not detect MCSF production in T cells [51], and to our knowledge, neither production nor a physiological role for MCSF derived from circulating T cells has ever been demonstrated *in vivo*.

**Fig 3:**
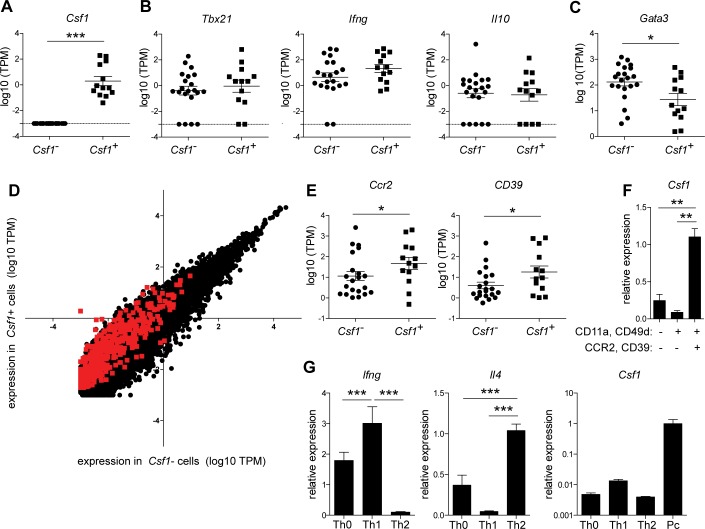
Transcriptional and phenotypic analysis of *Csf1* ^*+*^ CD4^+^ T cells. Antigen-experienced CD4^+^ T cells were sorted from blood 6 d.p.i. and subjected to single-cell RNA-Seq (n = 35 cells). (A-C) RNA-Seq measurements of the indicated transcripts in individual *Csf1* ^*+*^ and *Csf1* ^*-*^ T cells. TPM, transcripts per kilobase million. Dashed line indicates limit of detection. (D) Scatter plot depicting expression of all detected transcripts in *Csf1* ^*+*^ versus *Csf1* ^*-*^ cells. Red symbols indicate differentially expressed transcripts (FDR < 5%). (E) Expression of the indicated transcripts, as described for A-C. (F) *Csf1* expression was assessed by qRT-PCR in antigen-experienced CD4^+^ T cells sorted into CCR2 and CD39 high and low populations. Antigen-naive (CD11a^-^ CD49d^-^) cells from infected mice were included for comparison. (G) Naive splenocytes were stimulated *in vitro* with plate-bound α-CD3 and α-CD28, either alone (Th0) or in conjunction with Th1- or Th2-polarizing cocktails. Expression of the indicated genes was assessed after 5 d by qRT-PCR. For *Csf1*, antigen-experienced CD4^*+*^ T cells isolated from an infected mouse 6 d.p.i. are included as a positive control (Pc); note log scale. In (F-G), representative results from one of two independent biological replicates are shown (n = 4 mice or wells per condition per replicate). *, p < 0.05; **, p < 0.01; ***, p < 0.001 by Mann-Whitney (A) or *t*-test (others).

In order to characterize the nature of *Csf1* expression in T cells, we performed single-cell transcriptional analysis on antigen-experienced CD4^+^ T cells from infected mice (**S1 Table**). Although MCSF levels in plasma increase through day 14 (**Fig 2H**), likely arising from multiple systemic sources, *Csf1* transcript was most readily detected by qRT-PCR in blood CD4^+^ T cells 6 d.p.i.; therefore, this timepoint was chosen for further RNA-Seq analysis. Moderate to abundant levels of *Csf1* transcript were detected in 37% of the cells analyzed (13 out of 35), whereas no *Csf1* was detected in the remaining cells (**Fig 3A**). Single-cell sequencing results must be interpreted with caution, as transcript levels may fluctuate significantly within a cell over time due to the stochastic nature of gene expression [52]; in addition, technical limitations of the technique introduce stochasticity in transcript detection [53]. Nevertheless, the absence of *Csf1* transcript in a majority of CD4^+^ T cells leads us to favor the hypothesis that *Csf1* expression is restricted to a subset of activated cells, in which it is strongly upregulated.

T helper cells are traditionally classified into distinct lineages, each with a defining transcription factor and cytokine profile [54]. We examined whether *Csf1* ^*+*^ CD4^+^ T cells expressed hallmarks of any of the canonical T helper subsets. At the time-point sampled (6 d.p.i.), the CD4^+^ T cell response to *P*. *chabaudi* consists primarily of Th1-polarized cells [24,28]. Consistent with this, we found that nearly all *Csf1* ^*+*^ cells also expressed *Tbx21*, encoding the Th1 lineage-defining transcription factor TBET, and all expressed the canonical Th1 cytokine IFN-γ. Most *Csf1* ^*+*^ cells also expressed *Il10*, which is commonly co-expressed with IFN-γ in *Plasmodium*-exposed subjects [55–57] (**Fig 3B**). Expression of the Th2 transcription factor *Gata3* was significantly lower in *Csf1* ^*+*^ T cells than in *Csf1* ^*-*^ cells (**Fig 3C**); we did not detect the Th2 cytokines *Il4*, *Il5*, or *Il13*, or the Th17 markers *Rorc* or *Il17*, in any cell, regardless of *Csf1* expression. Thus, *Csf1* ^*+*^ cells express signature genes of Th1 cells, but not Th2 or Th17 cells.

Next we were interested in whether *Csf1* ^*+*^ T cells had a transcriptional signature that distinguished them from *Csf1* ^*-*^ cells. Although the vast majority of transcripts detected did not vary significantly between *Csf1* ^*-*^ and *Csf1* ^*+*^ cells, we identified a cluster of approximately 400 differentially expressed genes, representing 2.7% of detected transcripts (**Fig 3D** and **S2 Table**). This cluster included genes for several cell surface receptors that we subsequently examined by flow cytometry, choosing markers based on the availability of commercial antibodies as well as an expression pattern that would yield distinct populations for further transcriptional analysis. *Ccr2*, which encodes a chemokine receptor, and *Entpd1*, encoding the ectonuclease CD39, were both upregulated in *Csf1* ^*+*^ cells relative to *Csf1* ^*-*^ cells (**Fig 3E**). Therefore we sorted CCR2^hi^ CD39^hi^ antigen-experienced CD4^+^ T cells from infected mice and used qRT-PCR to compare their *Csf1* expression with CCR2^-^ CD39^-^ cells (gating strategy,**S2B Fig**). Whereas *Csf1* transcript was nearly undetectable in both antigen-naive cells and antigen-experienced CCR2^-^ CD39^-^ cells, it was readily detected in antigen-experienced CCR2^hi^ CD39^hi^ cells (**Fig 3F**). Virtually no CCR2^hi^ CD39^hi^ cells were observed within the antigen-naïve CD4^+^ T cell population, excluding the possibility that *Csf1* expression is linked to CCR2/CD39 expression independently of antigen experience. These results validate our RNA-Seq data at the level of protein expression and suggest that *Csf1* is expressed by a subset of CD4^+^ T cells enriched for a distinct surface phenotype. We did not observe *Csf1* expression in CD4^+^ T cells cultured *ex vivo* with either Th1- or Th2-polarizing cytokines (**Fig 3G),** even upon restimulation **(S4 Fig**), suggesting that additional signals provided in the context of *in vivo* infection are required to upregulate *Csf1*. Robust induction of the canonical cytokines *Ifng* and *Il4* in these Th1 and Th2 cultures, respectively, served as a control to confirm effective polarization (**Fig 3G**). Altogether, our data indicate that while *Csf1*-producing CD4^+^ T cells share most features with canonical Th1 cells, they possess some distinct transcriptional and surface characteristics that may be indicative of a specialized T helper phenotype or, alternatively, a particular activation state within the Th1 subset.

### MCSF drives macrophage proliferation and restricts parasitemia during *P*. *chabaudi* infection

**Fig 4:**
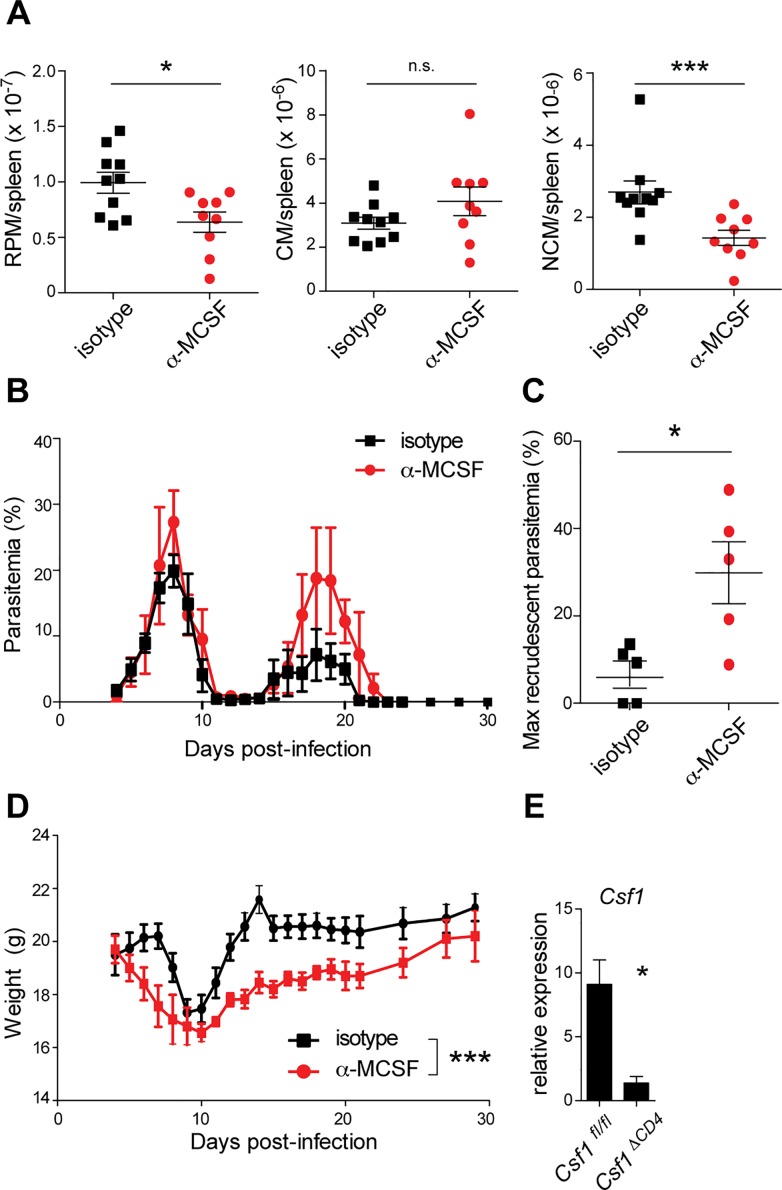
MCSF promotes myeloid expansion and parasite restriction during *P*. *chabaudi* infection. Infected mice were treated with α-MCSF or an isotype control antibody daily from 3–13 d.p.i. (A) The indicated myeloid subsets were enumerated in spleen on day 14 (n = 10 mice per group, pooled from two independent biological replicates). Parasitemia (B, C) and weight (D) were monitored. (E) *Csf1* expression was assessed by qRT-PCR in blood CD4^+^ T cells from mice of the indicated genotypes 6 d.p.i. In (B, D, E), mean +/- SEM is shown (n = 5 (B, D) or 4 (E) mice per group). In (C), maximum recrudescent parasitemias of individual mice are depicted. In (B-D), representative results from one of two biological replicates are shown. *, p < 0.05; ***, p < 0.001 by Mann-Whitney test. n.s., not significant.

After detecting inducible expression of MCSF transcript and protein in CD4^+^ T cells, we turned to the question of whether this cytokine indeed promoted myeloid expansion in the context of *Plasmodium* infection by administering an MCSF-blocking antibody systemically from 3 to 13 d.p.i. We chose this time frame to immediately precede the time when myeloid numbers peak (day 14; **Fig 1B**) as well as to overlap with the observed rise of MCSF levels in the plasma (**Fig 2H**), keeping in mind that that local production of MCSF in the tissues might well increase before elevated systemic MCSF levels could be measured [9,10]. After the blockade, mice treated with α-MCSF had significantly fewer splenic RPMs and NCMs on day 14 than mice treated with an irrelevant isotype control antibody (**Fig 4A**), demonstrating a role for systemic MCSF in myeloid expansion in the spleen during *P*. *chabaudi* infection. Numbers of blood monocytes were not significantly affected by MCSF blockade (**S5 Fig**).

Since macrophages are critical for control of parasitemia during the resolution phase of infection (**Fig 1D**) and disruption of MCSF signaling reduces macrophage numbers during this window (**Fig 4A**), we hypothesized that MCSF blockade would also result in increased parasite burden. Indeed, although it did not alter parasitemia during the acute phase, MCSF blockade significantly increased parasite recrudescence during the resolution phase of infection (**Fig 4B and 4C**), coincident with the peak of myeloid expansion in control mice. Additionally, mice treated with α-MCSF exhibited poor recovery from infection-induced weight loss, relative to mice treated with an isotype control antibody (**Fig 4D**). These data indicate that MCSF-driven myeloid proliferation is important for controlling parasite replication and limiting host morbidity. The fact that MCSF blockade did not affect acute parasitemia suggests that pre-existing macrophages and/or monocytes are sufficient to control the initial peak of infection, whereas expanded numbers of myeloid cells are required to suppress parasitemia as the infection persists into the resolution phase. In the same vein, systemic phagocyte depletion (**Fig 1C and 1D**) results in a more severe phenotype than MCSF blockade (Fig **4B and 4C**), which does not completely ablate the myeloid compartment but only reduces its numbers (**Fig 4A**).

### CD4^+^ T cell-derived MCSF contributes to control of infection

**Fig 5:**
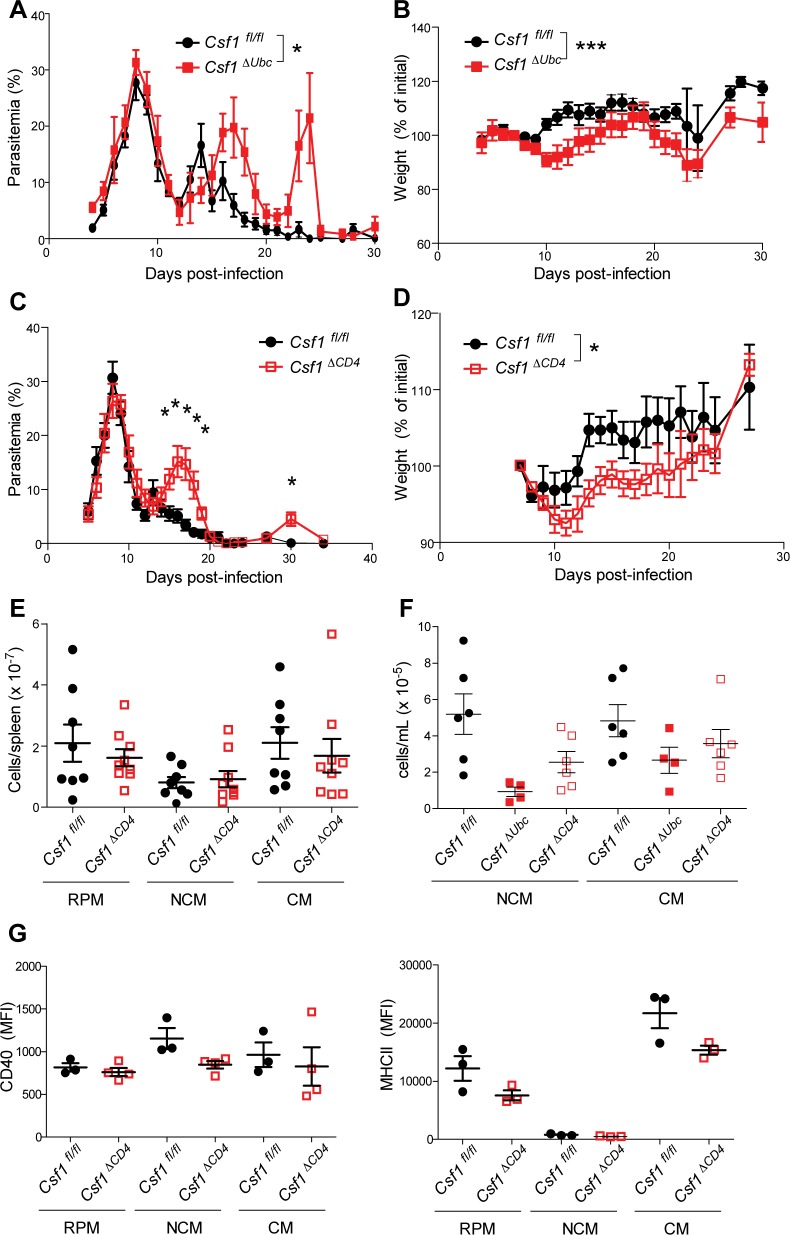
MCSF from CD4^+^ T cells is important for control of infection. (A, B) Control mice (*Csf1* ^*fl/fl*^) and mice that systemically delete *Csf1* (*Csf1* ^*Δ*Ubc^) were infected with *P*. *chabaudi* one month after induction of *Csf1* deletion, and parasitemia (A) and weight (B, normalized to starting weight) were monitored (n = 14 *Csf1* ^*fl/fl*^ and 9 *Csf1* ^*Δ*Ubc^ mice pooled from two independent experiments). (C, D) Parasitemia and weight loss following infection of control mice (*Csf1* ^*fl/fl*^) and mice with selective deletion of *Csf1* in CD4^+^ cells (*Csf1* ^*Δ*CD4^) (n = 12 *Csf1* ^*fl/fl*^ and 11 *Csf1* ^*Δ*CD4^ mice pooled from two biological replicates). (E) The indicated myeloid subsets were quantified in spleens from *Csf1* ^*fl/fl*^ and *Csf1* ^*Δ*CD4^ mice 14 d.p.i. Results are pooled from two independent experiments. (F) Blood monocyte numbers were measured in the indicated genotypes 14 d.p.i. (G) Expression (MFI) of CD40 (left) and MHCII (right) was measured on the indicated splenic myeloid subsets 14 d.p.i. Means +/- SEM are depicted in all graphs. In (E-G), each symbol represents an individual mouse. *, p < 0.05 by Mann-Whitney (A, D) or *t*-test (C); ***, p < 0.001 by Mann-Whitney test.

To test directly whether MCSF derived from CD4^+^ T cells is important for proliferation of myeloid cells and restriction of parasite burden, we generated mice that inducibly delete *Csf1* specifically in CD4^+^ cells (*Cd4*::*CreERT2*; *Csf1* ^*fl/fl*^, referred to hereafter as *Csf1* ^*ΔCD4*^). To induce *Csf1* deletion, *Csf1* ^*ΔCD4*^ mice and *Csf1* ^*fl/fl*^ littermate controls were fed tamoxifen chow beginning one month prior to infection and continuing through the duration of each experiment; disruption of *Csf1* expression in CD4^+^ T cells was confirmed by RT-qPCR after infection (**Fig 4E**). For comparison, we also generated and infected *Ubc*::*CreERT2*; *Csf1* ^*fl/fl*^ mice (referred to as *Csf1* ^*ΔUbc*^), which systemically delete *Csf1* upon tamoxifen treatment through expression of Cre recombinase under control of the ubiquitin promoter. After one month of tamoxifen treatment, monocyte and RPM numbers were significantly diminished in the spleens of naïve *Csf1* ^*ΔUbc*^ mice, consistent with the established role of systemic MCSF in maintenance of tissue-resident myeloid cells [10]. In contrast, *Csf1* ^*ΔCD4*^ mice were unaffected, indicating that CD4^+^ T cell-derived MCSF is not required to maintain myeloid cell numbers at baseline (**S6 Fig**). Consistent with the effects of MCSF blockade (**Fig 4A–4D**), *Csf1* ^*ΔUbc*^ mice exhibited significantly higher recrudescent parasitemia (**Fig 5A**) and lost more weight (**Fig 5B**) than control *Csf1* ^*fl/fl*^ mice. Importantly, selective deletion of *Csf1* in CD4^+^ cells also resulted in significantly higher parasite burdens (**Fig 5C**) and delayed recovery from weight loss (**Fig 5D**) during the resolution phase of infection, directly demonstrating a role for MCSF derived from CD4^+^ cells in control of *Plasmodium* infection. In addition to ubiquitous strong expression on CD4^+^ T cells, CD4 is expressed on a fraction of murine dendritic cells [58] and thymic macrophages [59]; however, given the lack of reported *Csf1* expression in these specific myeloid populations (www.immgen.org) and their relatively low representation within their respective leukocyte subsets, we consider it unlikely that they are responsible for the phenotype observed in *Csf1* ^*ΔCD4*^ mice. Moreover, previous characterization of the *Cd4*::*CreERT2* transgenic mouse line revealed high specificity for peripheral CD4^+^ T cells, with little to no recombination of floxed genes in CD11b^+^ myeloid cells despite reported expression of CD4 [60]. Thus we conclude that MCSF from CD4^+^ T cells contributes to control of parasite burden and host recovery during the resolution phase of *P*. *chabaudi* infection.

### CD4^+^ T cell-derived MCSF regulates expansion and activation of specific myeloid subsets

We next quantified myeloid populations in the spleens of infected *Csf1* ^*ΔCD4*^ mice 14 d.p.i. In addition to coinciding with the peak of myeloid expansion in wild-type mice (**Fig 1B**), this timepoint falls just prior to the greatest observed differences in parasitemia between *Csf1* ^*fl/fl*^ and *Csf1* ^*ΔCD4*^ mice (**Fig 5C**); by examining myeloid cells at this time, we hoped to uncover differences that would lie upstream of the divergent parasitemias observed on days 15–20, and to avoid examining phenotypes that might arise due to differences in parasite burden, rather than directly resulting from CD4-specific deletion of *Csf1*. Having observed increased parasitemia in mice lacking CD4^+^ T cell-specific expression of MCSF, we expected to find decreased myeloid expansion in the spleens of these mice. Surprisingly, however, numbers of splenic RPMs, CMs, and NCMs were intact in infected *Csf1* ^*ΔCD4*^ mice (**Fig 5E**). Combined with the baseline defects in mice that systemically delete *Csf1* (**S6 Fig**) as well as the MCSF blockade results (**Fig 4A**), these data indicate that MCSF from other sources, but not from CD4^+^ T cells, is required to stimulate splenic myeloid expansion during *P*. *chabaudi* infection.

We therefore searched for alternative mechanisms by which CD4^+^ T cell-derived MCSF might promote parasite restriction. First, we hypothesized that T cell-derived MCSF might support proliferation of myeloid subsets other than those we had previously examined. Consistent with this hypothesis, we measured diminished numbers of NCMs in the blood of infected *Csf1* ^*ΔCD4*^ mice relative to *Csf1* ^*fl/fl*^ controls 14 d.p.i. (**Fig 5F**). The defect in NCM numbers was milder in *Csf1* ^*ΔCD4*^ mice than in *Csf1* ^*ΔUbc*^ mice, indicating that MCSF derived both from CD4^+^ T cells and T cell-independent sources contributes to expansion of blood monocytes during infection (**Fig 5F**).

Additionally, we tested the hypothesis that MCSF from CD4^+^ T cells might promote activation of myeloid cells, as MCSF has been reported to do *in vitro* [9]. To accomplish this, we measured levels of activation markers on splenic myeloid subsets in infected mice. Interestingly, there was a trend toward lower expression of MHCII on RPMs and CMs from infected *Csf1* ^*ΔCD4*^ mice compared to infected *Csf1* ^*fl/fl*^ controls, while NCMs exhibited lower levels of CD40 (**Fig 5G**). However, these effects were modest and not statistically significant, similar to the effects of CD4^+^ cell-derived MCSF on monocyte numbers (**Fig 5F**), leading us to examine additional myeloid populations for differences that might explain the poor control of parasitemia observed in *Csf1* ^*ΔCD4*^ mice.

### A role for MCSF-dependent CD169^+^ macrophages in control of *P*. *chabaudi*

**Fig 6:**
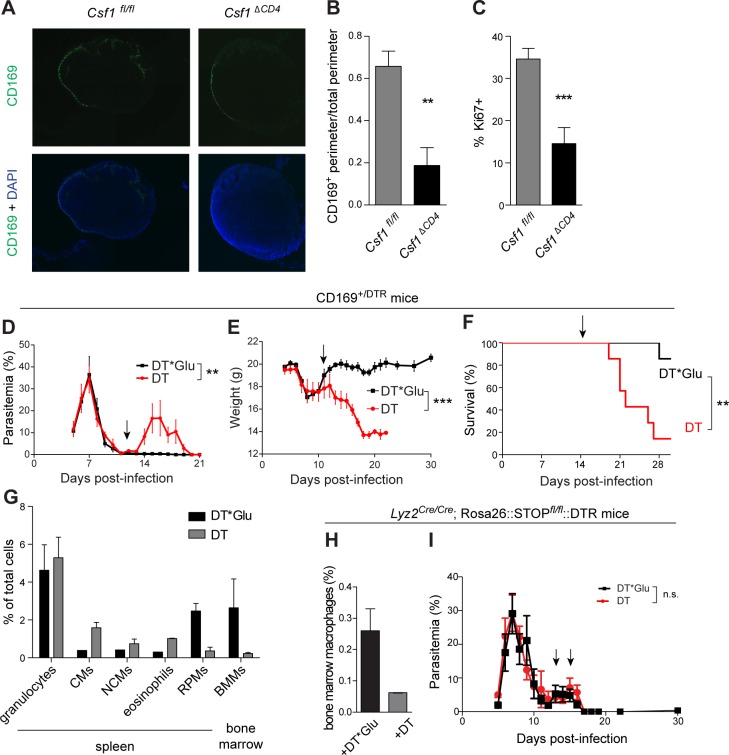
A role for CD169^+^ macrophages in restriction of *Plasmodium*. (A) Immunofluorescent labeling for the macrophage marker CD169 on mesenteric lymph nodes excised from the indicated mice 14 d.p.i. Representative sections are shown. (B) The fraction of each lymph node section perimeter lined with CD169^+^ cells, relative to the total section circumference, was quantified. (C) Lymph node sections from mice of the indicated genotypes were obtained 14 d.p.i. and co-labeled with antibodies to CD169 and Ki67. The percentage of CD169^+^ cells that were also Ki67^+^ was quantified. In B and C, graphs depict mean + SEM (n = 3 mice per group with 4 technical replicates for each mouse). **, p < 0.01; ***, p < 0.001 by *t*-test. (D-G) CD169^+/DTR^ mice were infected with *P*. *chabaudi* and treated with diphtheria toxin (DT; n = 7) or a catalytically inactive mutant (DT*Glu; n = 6) to deplete CD169^+^ cells 12 d.p.i. (arrows). Representative results from one of two independent experiments are shown. Parasitemia (D), weight (E), and survival (F) were monitored. (G) The indicated myeloid frequencies were assessed in infected CD169^+/DTR^ mice 24 h after administration of DT. (H, I) *Lyz2* ^*Cre/Cre*^; Rosa-DTR mice were infected and treated with DT (n = 5) or DT*Glu (n = 6) 13 and 15 d.p.i. (arrows) to deplete Lyz2^+^ cells. Results are representative of three independent experiments. Depletion of bone marrow macrophages was confirmed by flow cytometry (H), and parasitemia was monitored (I). **, p < 0.01; ***, p < 0.001 by Mann-Whitney test. n.s., not significant.

The CD169^+^ macrophages of the spleen and lymph nodes have been shown to play critical roles in the capture and clearance of blood- and lymph-borne pathogens and other antigens [61,62], but they are difficult to analyze by flow cytometry due to their fragility [63]. We therefore performed immunofluorescence microscopy to examine the effects of CD4^+^ T cell-derived MCSF on CD169^+^ macrophage populations, including splenic marginal metallophillic macrophages (MMMs) and the subcapsular sinus macrophages (SCSMs) in the lymph nodes. Consistent with previous reports that show near-complete disappearance of CD169^+^ macrophages in the spleens of mice infected with *P*. *chabaudi* [64,65], we detected few CD169^+^ macrophages in the spleens of either *Csf1* ^*fl/fl*^ or *Csf1* ^*ΔCD4*^ mice 14 d.p.i. However, in lymph nodes, there were significant differences in the distribution of CD169^+^ macrophages between *Csf1* ^*fl/fl*^ and *Csf1* ^*ΔCD4*^ mice. In the absence of a straightforward method such as flow cytometry to assess absolute numbers of CD169^+^ macrophages [63], we quantified the fraction of each lymph node perimeter that was lined with CD169^+^ cells (**Fig 6A and 6B**). Strikingly, in *Csf1* ^*ΔCD4*^ mice, a significantly smaller fraction of each lymph node was lined with CD169^+^ macrophages, indicating a role for CD4^+^ T cell-derived MCSF in supporting the survival or expansion of these cells (**Fig 6B**).

To further distinguish whether CD4^+^ T cell-derived MCSF affects proliferation of lymph node macrophages, we also assessed the fraction of CD169^+^ macrophages that were Ki67^+^ by immunofluorescence microscopy in lymph node sections from *Csf1* ^*fl/fl*^ and *Csf1* ^*ΔCD4*^ mice (**S7 Fig**). Quantification of Ki67^+^ cells revealed significantly fewer proliferating macrophages in lymph nodes from *Csf1* ^*ΔCD4*^ mice (**Fig 6C**). Thus, MCSF from CD4^+^ T cells is important for promoting proliferation of CD169^+^ macrophages in the lymph nodes of infected mice.

CD169^+^ macrophages have not previously been examined for involvement in control of *P*. *chabaudi* infection, but a recent study demonstrated that systemic depletion of CD169^+^ macrophages increased tissue sequestration of parasites, morbidity, and mortality in a model of experimental cerebral malaria (ECM) employing the pathogen *P*. *berghei* ANKA in ECM-resistant Balb/C mice. In light of this study and the immunofluorescence data, we tested whether CD169^+^ macrophages are important for control of parasitemia during the resolution phase of *P*. *chabaudi* infection in B6 mice, when MCSF production by T cells is most critical for restriction. We infected transgenic CD169^+/DTR^ mice, which express the diphtheria toxin (DT) receptor under control of the CD169 promoter [66]. Treatment of these mice with DT results in efficient depletion of splenic MMMs ([66,67] and **S8 Fig**) and lymph node SCSMs [67,68]. Mice depleted of CD169^+^ cells 12 d.p.i. developed significantly higher parasitemia (**Fig 6D**), weight loss (**Fig 6E**), and mortality (**Fig 6F**) relative to controls treated with a catalytically inactive point mutant (DT*Glu), indicating a role for these macrophages in control of *P*. *chabaudi*. To exclude off-target effects of DT treatment, we examined frequencies of additional myeloid subsets. In addition to MMMs and SCSMs, DT treatment in CD169^+/DTR^ mice also resulted in depletion of bone marrow macrophages, which express CD169 [69] (**Fig 5G)**; however, using a separate transgenic mouse model, in which the DT receptor is expressed under control of the *Lyz2* promoter (*Lyz2* ^*Cre/Cre*^; Rosa26::STOP^fl^/^fl^::DTR) [70], we found that depletion of bone marrow macrophages, as confirmed by flow cytometry (**Fig 5H**), in itself had no effect on parasitemia (**Fig 5I**). In addition, although DT treatment diminished RPM frequencies in CD169^+/DTR^ mice (**Fig 5G**), we have previously shown that RPMs are not required for control of *P*. *chabaudi* [71]. Therefore, these results are most consistent with a model in which CD4^+^ T cell-derived MCSF promotes the survival or proliferation of CD169^+^ SCSMs, which contribute critically to parasite control and host survival.

**Fig 7:**
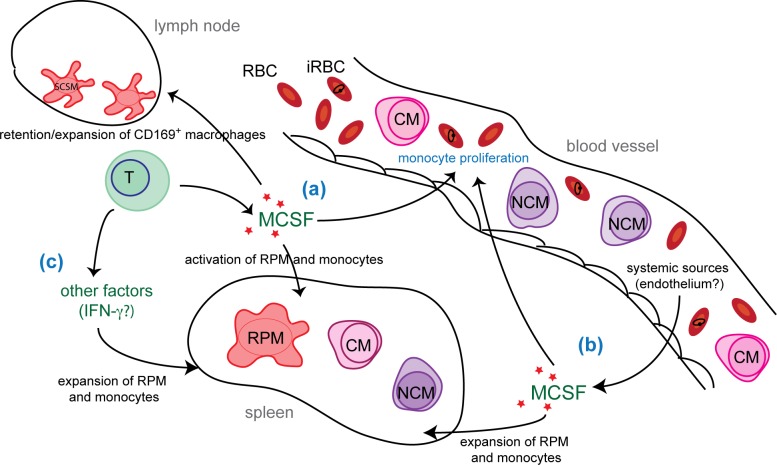
Multiple roles for CD4^+^ T cells and MCSF in regulation of myeloid cells during *Plasmodium* infection. (A) CD4^+^ T cell-derived MCSF positively regulates the abundance of CD169^+^ macrophages in the lymph nodes, and contributes to proliferation of blood monocytes and activation of red pulp macrophages (RPM) and classical and nonclassical monocytes (CM, NCM) in the spleen. (B) MCSF derived from non-CD4^+^ T cell sources (e.g., endothelial cells) drives expansion of myeloid cells in both the spleen and the blood. (C) CD4^+^ T cells also promote splenic myeloid expansion independently of MCSF, perhaps through production of IFN-γ or other cytokines. RBC, red blood cell. iRBC, infected RBC.

Together with flow cytometry analyses (**Figs 4A, 5E and 5F**), the above experiments reveal that multiple sources of MCSF drive myeloid expansion during *P*. *chabaudi* infection. T cell-independent sources are most important for proliferation of the splenic myeloid subsets examined, whereas T cell-derived MCSF is critical for maintenance and proliferation of CD169^+^ macrophages in the lymph nodes, as well as contributing modestly to expansion and activation of some additional subsets in the blood and spleen. In addition, by comparing the phenotype of mice depleted of CD4^+^ T cells (**Fig 2B**) with mice simply lacking *Csf1* expression in CD4^+^ T cells (**Fig 5E**), we conclude that CD4^+^ T cells promote myeloid expansion through multiple mechanisms, only one of which is production of MCSF (**Fig 7**).

## Discussion

Previous publications have detected production of MCSF by cultured CD4^+^ T cells and in the special case of by decidual CD4^+^ T cells during pregnancy, but production by circulating T cells *in vivo* has never been demonstrated to our knowledge, and no physiological role has ever been assigned to CD4^+^ T cell-derived MCSF. In recent years, a number of tools have become available that have proved useful for dissecting this phenomenon *in vivo*. Here, using conditional knockout mice and sensitive methods for cell surface and transcriptional analysis, we have identified an important role for MCSF derived from CD4^+^ T cells in control of blood-stage *P*. *chabaudi*. This experimental malaria model has long been used to simulate uncomplicated infection with *P*. *falciparum*, the principal etiological agent of a human disease that caused 214 million new illnesses and ~438,000 deaths in 2015 [72]. Thus, in addition to elucidating a novel mechanism through which T cells regulate myeloid cells to restrict infection, this work provides insight into the immune correlates of protection from a devastating pathogen for which a successful vaccine has thus far proved elusive.

Because CD4^+^ T cells are such central mediators of myeloid cell activation, it makes sense that can they also regulate myeloid cell numbers by promoting proliferation and/or survival. Indeed, T cells likely stimulate expansion of the myeloid compartment through multiple mechanisms, depending on the context. During helminth infection, local IL-4-dependent proliferation of macrophages in the tissues has been shown to require an intact *Rag* locus, indicating a role for B and/or T lymphocytes [6,7]; it is plausible that in this model, T cells drive macrophage proliferation through production of IL-4. Further, T cells can produce Granulocyte-Macrophage CSF, which has been shown to regulate absolute leukocyte numbers in mice infected with *P*. *chabaudi* [3]. To these mechanisms we now propose to add the inducible production of MCSF, which we have shown stimulates not only expansion, but also activation of myeloid cells.

We demonstrated that selective deletion of *Csf1* in CD4^+^ T cells during infection results in significant reductions in the abundance and proliferation of CD169^+^ macrophages in the lymph nodes, as well as trends towards decreased numbers of some myeloid cells (blood NCMs) and diminished activation of other subsets (splenic RPMs, NCMs, and CMs). An alternative interpretation of our immunofluorescence data is that disruption of CD4^+^ T cell-derived MCSF results in diminished CD169 expression, rather than loss of CD169^+^ macrophages; however, in the absence of additional surface markers to positively identify SCSMs in lymph nodes, this possibility is difficult to test experimentally. It is not yet clear whether or how the more modest effects of CD4^+^ T cell-specific *Csf1* deletion on monocyte numbers and macrophage/monocyte activation contribute to the observed increases in parasitemia and morbidity in *Csf1* ^*ΔCD4*^ mice. However, our experiments in mice depleted of CD169^+^ cells clearly show an important role for this subset in control of *Plasmodium* parasitemia and host survival, suggesting that a primary function of CD4^+^ T cell-dependent MCSF during *P*. *chabaudi* infection is to support these cells. In contrast to some other macrophage populations, including the lymph node medullary macrophages and splenic RPMs, lymph node SCSMs are critically dependent on MCSF for their survival and proliferation [73]; perhaps this dependency makes them particularly vulnerable to disruption of a single source of MCSF.

CD4^+^ T cells are certainly not the only source of MCSF during *P*. *chabaudi* infection. Indeed, depletion of CD4^+^ T cells did not decrease plasma concentrations of MCSF in infected mice, indicating multiple redundant producers of this cytokine. However, our data demonstrate that CD4^+^ T cell-derived MCSF does play a nonredundant role in sustaining SCSM abundance and limiting recrudescent parasitemia. This may reflect a requirement for localized production of MCSF, which exists in the body not only as a soluble glycoprotein, but also in membrane-bound and proteoglycan forms, the latter of which contributes to circulating MCSF levels but may also preferentially accumulate in extracellular matrix [10]. We hypothesize that antigen-experienced CD4^+^ T cells in the blood and tissues may deliver MCSF directly to monocytes and macrophages, perhaps in conjunction with other signals, providing a stimulus that cannot be replaced by MCSF derived from other sources. Consistent with this hypothesis, T cells have been observed to interact closely with SCSMs in the lymph nodes of both naïve mice and those infected with *Toxoplasma*, an apicomplexan parasite related to *Plasmodium* [74,75]. Our data do not rule out the possibility that CD169^+^ macrophages other than SCSMs are the relevant population for control of parasitemia; for example, it may be that despite their small numbers, the MMMs that persist in infected spleens are nonetheless critical for restriction. In addition, it remains possible that CD4^+^ T cell-derived MCSF contributes to parasite control through yet another alternative mechanism, such as modulation of a myeloid population that we have not examined here.

Having discovered a population of activated CD4^+^ T cells that inducibly expresses *Csf1*, we must consider how these cells fit into the established T helper paradigm. Although recent demonstrations of T cell plasticity have begun to blur the lines between different Th subsets [76], we maintain that some key elements of a T cell lineage can be defined: individual Th lineages generally have unique master transcription factors, canonical cytokines, and chemokine receptors, and their phenotype is stable and self-reinforcing [77].

In this case, robust expression of *Tbx21* and overall transcriptional similarity to Th1 cells, which constitute the majority of CD4^+^ T cells at this stage of *Plasmodium* infection, support the hypothesis that *Csf1*-producing cells represent a specialized subclass of Th1 cells. On the other hand, we note that in a transcriptional profiling study of Th1, Th2, and Tfh cells polarized *in vitro*, the subsets differed significantly in expression of only 300–400 genes, similar to the number of genes that were differentially expressed between *Csf1* ^*+*^ and *Csf1* ^*-*^ cells in our analysis [78]. Further, we detected a number of transcription factors that were differentially expressed in *Csf1* ^+^ compared to *Csf1* ^*-*^ T cells (S2 Table); it may be that one or more of these acts in concert with TBET to exert a distinct transcriptional program in *Csf1* ^+^ cells. Indeed, one of the differentially expressed transcription factors in our dataset, *Bhlhe40*, was recently shown to drive GM-CSF expression in a fraction of Th1 and Th17 cells during experimental autoimmune encephalitis [79–81]. Previous studies on the gene expression profiles of CD4^+^ T cells polarized *in vitro* detected *Csf1* transcript in Th2 cells [82], and found no defect in *Csf1* expression in *Bhlhe40*-deficient CD4^+^ T cells [80]; however, given that we detected little *Csf1* expression in cells polarized *in vitro* relative to those isolated from infected mice, we hypothesize that these previous gene expression studies lack a robust positive control for *Csf1* expression, and instead are measuring relatively low levels of transcript that may not be physiologically meaningful.

Monocytosis is a common feature of malaria and several other chronic infections, such as tuberculosis and leishmaniasis [83,84], but its causes and significance have not been well characterized. This study elucidates one mechanism of myeloid proliferation and activation during malaria and demonstrates that expansion of macrophages and monocytes is critical for ongoing restriction of *Plasmodium* parasite growth. It will be of interest to determine whether a similar mechanism operates in other infectious settings in which CD4^+^ T cells cooperate with macrophages to limit microbial burden, and to dissect the signals required for MCSF induction. In addition, it remains to be seen whether CD4^+^ T cells inducibly produce MCSF in sterile disease settings, such as tumor microenvironments, in which macrophages and other myeloid cells play important roles.

## Materials and Methods

### Ethics statement

All animal experiments were conducted with the approval of the UCSF Institutional Animal Care and Use Committee (Protocol AN086391-03C) in accordance with the “Guide for the Care and Use of Laboratory Animals,” published by the National Research Council and endorsed by the NIH Office of Laboratory Animal Welfare.

### Mice

Mice were housed on a twelve hour light-dark cycle under specific pathogen free conditions. C57Bl/6 mice were from the National Cancer Institute. *Cd4*::*CreERT2* and *Ubc*::*CreERT2* mice (Jackson) were crossed to *Csf1* ^*fl/fl*^ mice [85] (kindly provided by S. Abboud-Werner, University of Texas Health Science Center) to generate hemizygous *CreERT2*; *Csf1* ^*fl/fl*^ mice. CD169^+/DTR^ mice were kindly provided by J. Cyster (UCSF) and M. Tanaka (RIKEN Research Center for Allergy and Immunology) [66]. *Lyz2* ^*Cre/Cre*^ mice [70] were bred to Rosa26::STOP^fl^/^fl^::DTR mice in-house (both Jackson). Female 8–12 week old mice were used for infections; littermate controls were used for all experiments with floxed and DT-treated mice. To induce deletion of floxed *Csf1*, mice were fed tamoxifen chow (Envigo) *ad libitum* beginning 1 month prior to infection and through the duration of each experiment.

### Infections

Mice were infected with 10^6^ *Plasmodium chabaudi* AS (MRA-429; MR4 Stock Center) parasitized RBCs, and parasitemia was monitored by thin film blood smear as described [86]. Where noted, mice were injected i.p. with the following: 300 μg α-CD4 (GK1.5) or isotype control (LTF2); 500 μg α-MCSF (5A1) or isotype control (HRPN) (all BioXCell); or 300 μL liposomes loaded with PBS or clodronate at neutral pH (FormuMax Scientific). To deplete CD169^+^ cells, heterozygous CD169^+/DTR^ mice were treated 12 d.p.i. with a single i.p. dose (80 ng/g) of DT (Sigma D0564) or a catalytically inactive point mutant (DT*Glu; Sigma D2189). To deplete Lyz2^+^ cells, including bone marrow macrophages, *Lyz2* ^*Cre/Cre*^; Rosa26::STOP^fl^/^fl^::DTR mice were treated with 500 ng DT or DT*Glu on d 13 and d 15 post-infection.

### Flow cytometric analysis

Blood was obtained by cardiac puncture or submandibular bleed; spleens were excised and homogenized after euthanasia according to approved protocols. Following RBC lysis in ACK buffer, samples were blocked, labeled with antibodies, and analyzed on an LSR II (BD) to assess myeloid cell frequencies. Intracellular Ki67 levels were measured using the Fixation and Permeabilization Buffer Set (eBioscience) and were compared to cells labeled with an isotype control antibody. Antibodies are listed in **S3 Table**. The Annexin V Apoptosis Detection Kit (eBioscience) was used with propidium iodide to quantify apoptotic cells. For EdU labeling, mice were injected i.p. with 750μg EdU 3 h prior to sacrifice, and the Click-It EdU Kit (Thermo Fisher) was used to detect EdU in splenocytes according to the manufacturer’s protocol.

Myeloid cell definitions were as follows: red pulp macrophages and bone marrow macrophages (Ly6G^-^ Ly6C^-^ CD11b^lo^ F4/80^+^); nonclassical monocytes (Ly6G^-^ Ly6C^-^ CD11b^+^ F4/80^int^ SSC^lo^); classical monocytes (Ly6G^-^ Ly6C^++^ CD11b^+^ F4/80^int^ SSC^lo^) (**S1A Fig**). Additional labeling with CD115 and CD68 was performed to confirm the identities of monocyte and macrophage populations (**S1B and S1C Fig**). Absolute cell numbers were quantified in blood prior to RBC lysis using the Guava Viacount assay (EMD Millipore) and in RBC-lysed spleen samples using a hemocytometer.

### Microarray analysis

Antigen-experienced T cells (**S2A Fig**, [42,43]) were isolated from wild-type mice 6 d.p.i. by double-sorting to high purity on a FACSAria (BD). RNA was isolated using the RNAqueous Micro kit (Ambion) and amplified (Amino Allyl MessageAmp II kit, Life Technologies) to generate amino allyl incorporated amplified RNA (aaRNA). aaRNA was coupled to Cy3 dye (GE Healthcare Life Sciences) and hybridized overnight to a SurePrint G3 Mouse Gene Expression 8x60K microarray (Agilent), which was washed and scanned per manufacturer’s instructions. Raw intensities were extracted using Feature Extraction software (Agilent) and quantile normalized using Limma [87]. Differentially expressed genes were identified using Significance Analysis for Microarrays (SAM) [88]. Complete microarray data can be accessed in the Gene Expression Omnibus database (GEO; http://www.ncbi.nlm.nih.gov/geo/) under accession GSE81196.

### Quantitative RT-PCR

Blood cells were labeled with antibodies (**S3 Table** and flow cytometry methods above) and sorted on a FACSAria (BD) directly into lysis buffer. RNA was isolated using the RNAqueous Micro Kit (Ambion) and reverse-transcribed into cDNA using Superscript III (Life Technologies) primed with dT(20)V. For microarray validation, cDNA was made from aaRNA with minor protocol modifications as described [89]. Quantitative PCR was performed in a Step One Plus RT PCR System (Applied Biosystems) using PerfeCTa 2x qPCR mix (Quanta). Transcript levels were normalized to levels of actin mRNA. Primer sequences are listed in **S4 Table**.

### ELISA

To examine MCSF production by T cells, CD11a^+^ CD49d^+^ CD4^+^ T cells were sorted on a FACSAria (BD) from infected mice 6 d.p.i. and cultured for 4 d in 96-well plates at 10^5^ cells/well with PMA (10 ng/mL) and ionomycin (1 μg/mL) (both Fisher). Cell-free supernatants were harvested for ELISA. Antigen-naive (CD11a^-^ CD49d^-^) CD4^+^ T cells and non-T cells (TCRβ^-^) were sorted and cultured as controls. For plasma measurements, blood was collected from the sub-mandibular vein into K_2_EDTA and centrifuged to separate cells from plasma, which was snap-frozen and stored at -80°C until analysis. MCSF was measured using the Murine M-CSF ELISA Development Kit (PeproTech) according to the manufacturer's protocol.

### Immunofluorescence

Mesenteric lymph nodes were obtained 14 d.p.i. and immediately frozen in Tissue-Tek OCT Compound (Sakura Finetek). 10μm sections were mounted, fixed in acetone, and labeled with FITC-conjugated α-CD169 (MOMA-1; ABD Serotec) followed by Alexa488-conjugated α-FITC (Jackson Immunoresearch). In some experiments, directly fluoroconjugated α-Ki67 (eBioscience) was also used. To examine MMMs, infected CD169^+/DTR^ mice were treated 12 d.p.i. with DT or DT*Glu as described above, and spleen sections were obtained 24 h later. Sections were labeled with antibodies to B220 (RA3.3A1/6.1, UCSF Monoclonal Antibody Core), CD169 (MOMA-1, AbD Serotec), and F4/80 (BM8, UCSF) to label B cells, MMMs, and RPMs, respectively. All samples were coverslipped with Vectashield Mounting Medium with DAPI (Vector) and visualized using an AxioCam HR camera on an AxioImagerM2 upright microscope.

To quantify CD169 labeling, lymph nodes were viewed in Image J (https://imagej.nih.gov/ij/) and the Segmented Lines tool was used to measure the length of the lymph node perimeter that labeled positively with α-CD169. This was divided by the total circumference of the lymph node, measured using the Segmented Lines tool on DAPI^+^ cells, to obtain a numerical value for the fraction of the lymph node capsule that was lined by CD169^+^ cells. Four technical replicates (i.e., individual tissue sections, separated by at least 20μm) were performed for each biological replicate (i.e., individual mouse).

### Single cell RNA-Seq

Antigen-experienced CD4^+^ T cells were isolated to high purity, using two consecutive rounds of FACS, from the blood of mice 6 d.p.i. Sorted cells were loaded onto a Fluidigm C1, captured, and processed into cDNA libraries following manufacturer protocols. Capture sites with zero or more than one cell were excluded from the libraries; libraries from 40 total cells were indexed, pooled into a single library, and sequenced on a HiSeq 2500 in high output mode. Reads were aligned using RSEM 1.2.22 and STAR 2.4.2a to GRCm38; samples contained an average depth of 3.9 million aligned reads, with 84.9% of reads aligning. Samples with fewer than 0.5 million aligned reads were excluded from further processing. For differential gene expression analysis, edgeR 3.4.2 was used to identify significantly differentially expressed genes between cells with *Csf1* expression of 0 TPM versus *Csf1* expression of > 1 TPM (FDR < 5%). All RNA-Seq data are available in GEO under accession GSE81197.

### *In vitro* T cell polarization

After RBC lysis, splenocytes from naive mice were incubated in plates coated with α-CD3 and α-CD28 (2.5 μg/mL each; UCSF Monoclonal Antibody Core) at a concentration of 10^6^ cells/mL in T cell media (RPMI + 10% FBS + 1mM sodium pyruvate + 50 U/mL penicillin + 50 U/mL streptomycin + 0.1% β-2-mercaptoethanol) alone or with the following polarization cocktails: for Th1, IL-12 (10 ng/mL, R&D) + α-IL-4 (10 μg/mL, Biolegend); for Th2, IL-4 (10 ng/mL, R&D) + α-IFN-γ (10 μg/mL, Biolegend). RNA was harvested after 5 d and processed for RT-qPCR as above. Where indicated, PMA and ionomycin were added after 5 d culture and samples were incubated for 6 h before RNA harvest. Simultaneously, splenocytes from a naïve mouse were plated in PMA + ionomycin for 6 h and processed for RNA along with cultured samples.

## Supporting Information

S1 Fig Gating strategy for myeloid cells. (**A**) Gating of different myeloid populations. A representative spleen sample is shown. (**B**) CD115 labeling to confirm monocyte identity of cells gated according to the scheme in (A). (**C**) RPMs gated as in A are CD115^+^ and CD68^+^, confirming identity as tissue macrophages.
(TIF)

S2 Fig Apoptosis of splenic myeloid populations in infected mice depleted of CD4^+^ T cells. Mice were infected with *P. chabaudi*, then depleted of CD4^+^ T cells 4 d.p.i. Controls were treated with an irrelevant isotype antibody. Frequencies of apoptotic cells were measured 14 d.p.i. by flow cytometric assessment of Annexin V and propidium iodide (PI) staining. Mean + SEM is shown (n = 3–5 mice per group). n.s., not significant. *, p < 0.05 by *t*-test.
(TIF)

S3 Fig Gating strategies for CD4+ T cells. (**A**) Antigen-experienced CD4^+^ T cells were defined as TCRβ^+^ CD4^+^ CD11a^+^ CD49d^+^. (**B**) Antigen-experienced cells, gated as described in (A), were further differentiated by CD39 and CCR2 expression. The double-hi and double-negative quadrants were sorted for comparison of *Csf1* expression by RT-qPCR.
(TIF)

S4 Fig *Csf1* expression in T cells following *in vitro* polarization and restimulation. Naïve splenocytes were cultured for 5 d on plates coated with anti-CD3 and anti-CD28 in the presence of Th1 and Th2-polarizing cocktails (see Methods). On day 5, cultures were stimulated for 6 h, RNA was harvested, and Csf1 transcript was measured by RT-qPCR, normalized to actin expression. Freshly harvested naïve splenocytes were also stimulated for 6 h along with cultures. Blood CD4^+^ antigen-experienced T cells sorted from a mouse infected 6 d with *P*. *chabaudi* (Pc) were used as a positive control for *Csf1* expression. Mean + SD is shown (n = 3 per group).
(TIF)

S5 Fig MCSF blockade does not affect blood monocyte levels. Infected mice were treated with anti-MCSF or an isotype control antibody daily from 3–13 d.p.i. Absolute numbers of classical (CMs) and nonclassical monocytes (NCMs) were assessed in the blood on day 14. Mean and SEM are shown (n = 5 per group).
(TIF)

S6 Fig Baseline myeloid frequencies in conditional *Csf1*-deficient mice. Mice of the indicated genotype were fed tamoxifen chow for one month to induce Cre-mediated deletion of *Csf1*. Absolute numbers of the indicated myeloid populations were quantified in the spleen. Graph depicts mean and SEM of 8 (*Csf* ^*fl/fl*^) or 4 (others) mice.
(TIF)

S7 Fig Ki67 labeling of CD169^+^ lymph node macrophages in conditional *Csf1*-deficient mice. Mice of the indicated genotypes were infected. Mesenteric lymph nodes were harvested 14 d.p.i. and labeled with antibodies to CD169 (green) and Ki67 (red) as well as with the nuclear dye DAPI (blue). White arrowheads indicate selected cells that co-label with CD169 and Ki67. Two representative images per genotype are shown.
(TIF)

S8 Fig Depletion of CD169^+^ macrophages in the spleens of CD169-DTR mice. CD169-DTR mice were infected and treated 12 d.p.i. with diphtheria toxin (DT) or a catalytically inactive point mutant (DT*Glu). Spleen sections were labeled with antibodies to CD169 (green), B220 (blue), and F4/80 (red) to label MZMs/MMMs, B cells, and RPMs, respectively. Representative images from at least three mice for each condition are shown.
(TIF)

S1 Table RNA-Seq expression data from single CD11a+ CD49d+ T cells sorted from mouse blood 6 d post-infection with *Plasmodium chabaudi*. Data are expressed as log(transcripts per kilobase of gene per million reads). Each column represents expression in a unique cell.
(XLSX)

S2 Table Genes differentially expressed in *Csf1+* and *Csf1-* antigen-experienced CD4+ T cells. Values are averaged from 22 Csf1- and 13 Csf1+ cells. Units are TPM (transcripts per kilobase of gene per million reads). Genes are ordered by the magnitude of the difference between Csf1+ and Csf1- cells.
(XLSX)

S3 Table Flow cytometry antibodies used in this study. (DOCX)

S4 Table Quantitative PCR primers used in this study. (DOCX)
