# Correction: Macrophage Colony Stimulating Factor Derived from CD4^+^ T Cells Contributes to Control of a Blood-Borne Infection

**DOI:** 10.1371/journal.ppat.1006192

**Published:** 2017-02-01

**Authors:** Mary F. Fontana, Gabrielly L. de Melo, Chioma Anidi, Rebecca Hamburger, Chris Y. Kim, So Youn Lee, Jennifer Pham, Charles C. Kim

A reference is omitted from the first sentence of the third paragraph under the subheading “A role for MCSF-dependent CD169+ macrophages in control of *P*. *chabaudi*” in the Results section. The sentence should read: CD169+ macrophages have not previously been examined for involvement in control of P. chabaudi infection, but a recent study demonstrated that systemic depletion of CD169+ macrophages increased tissue sequestration of parasites, morbidity, and mortality in a model of experimental cerebral malaria (ECM) employing the pathogen P. berghei ANKA in ECM-resistant Balb/C mice (Gupta et al., 2016).

The reference is: Gupta et al., 2016, Cell Reports 16, 1749–1761.

## References

[1] Fontana MF, de Melo GL, Anidi C, Hamburger R, Kim CY, Lee SY, et al (2016) Macrophage Colony Stimulating Factor Derived from CD4^+^ T Cells Contributes to Control of a Blood-Borne Infection. PLoS Pathog 12(12): e1006046 doi: 10.1371/journal.ppat.1006046 27923070 10.1371/journal.ppat.1006046 PMC5140069

