# Supplementary figures and images for: Macrophage Colony Stimulating Factor Derived from CD4+ T Cells Contributes to Control of a Blood-Borne Infection
Source: PLoS Pathog. 2016 Dec 6;12(12):e1006046. doi: 10.1371/journal.ppat.1006046 (PMC5140069; doi:10.1371/journal.ppat.1006046)

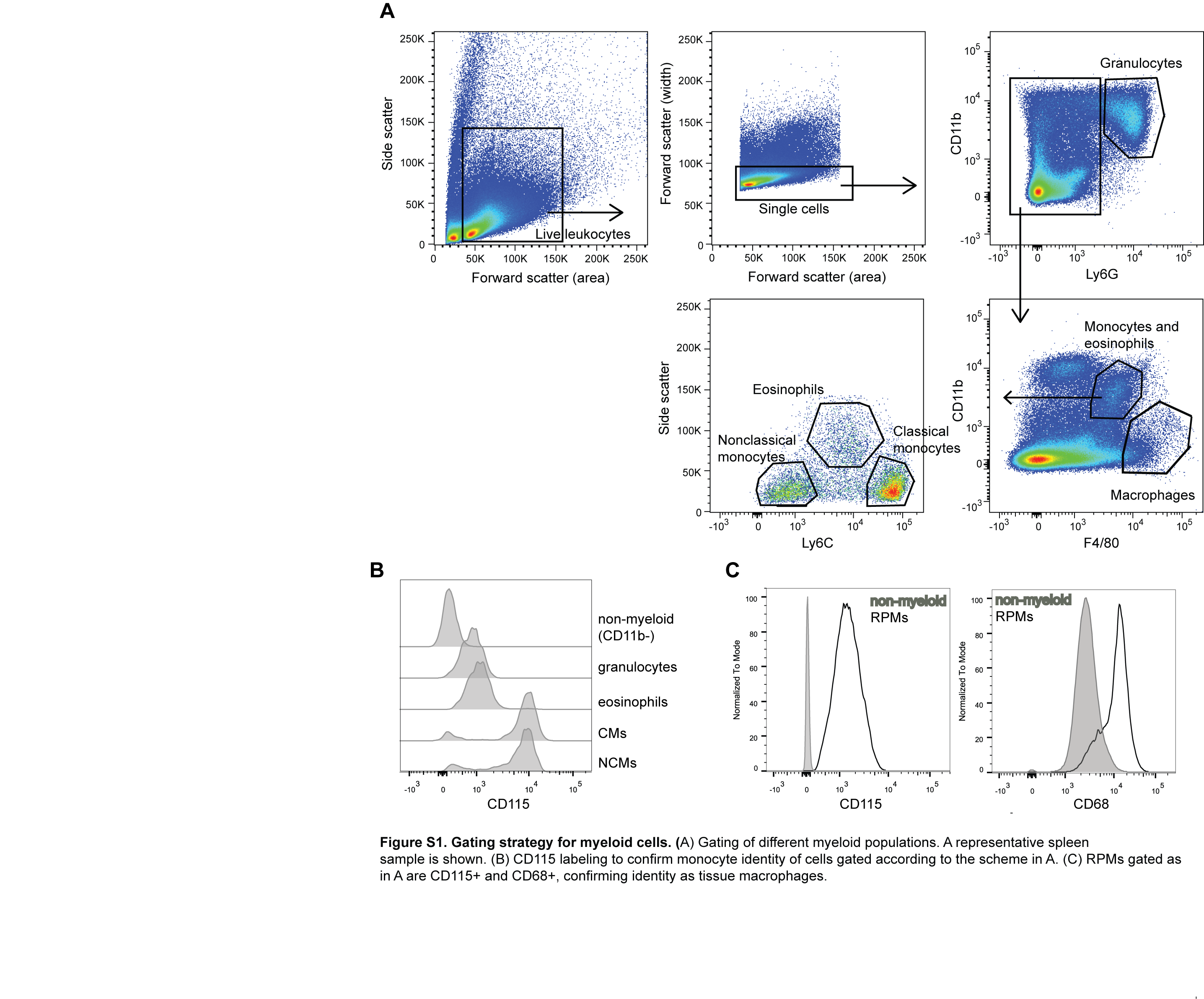

Supplement: S1 Fig — (A) Gating of different myeloid populations. A representative spleen sample is shown. (B) CD115 labeling to confirm monocyte identity of cells gated according to the scheme in (A). (C) RPMs gated as in A are CD115+ and CD68+, confirming identity as tissue macrophages. (TIF) [file ppat.1006046.s001.tif]

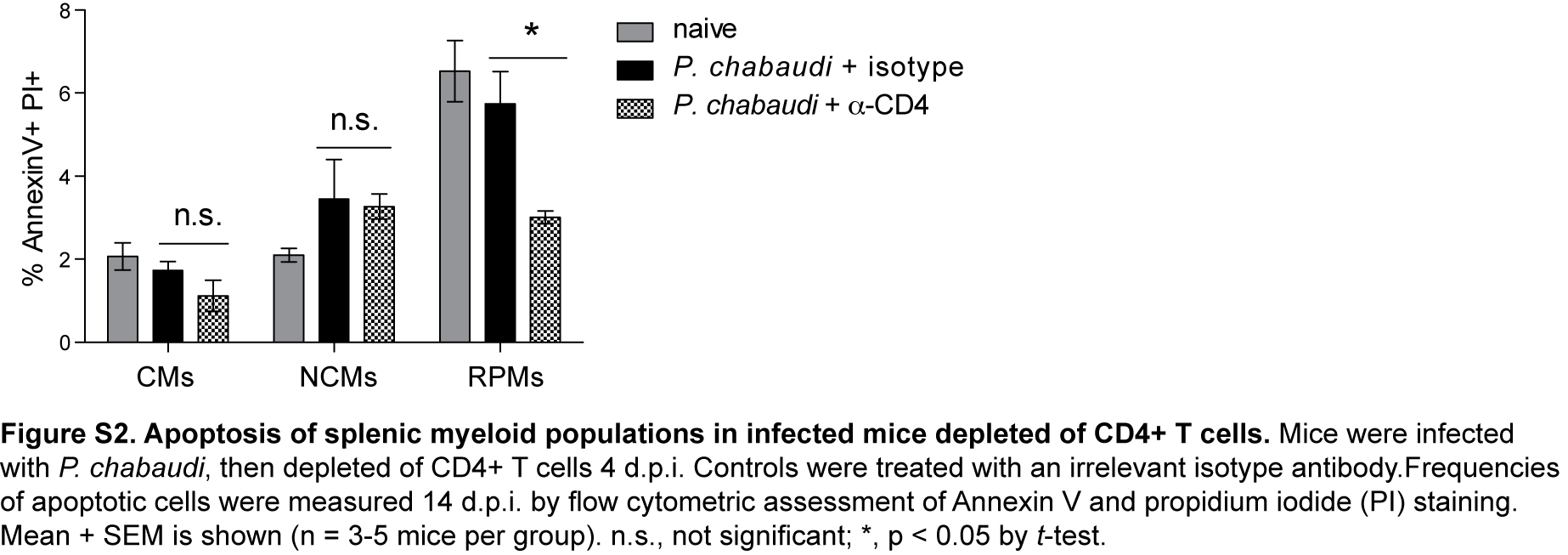

Supplement: S2 Fig — Mice were infected with P. chabaudi, then depleted of CD4+ T cells 4 d.p.i. Controls were treated with an irrelevant isotype antibody. Frequencies of apoptotic cells were measured 14 d.p.i. by flow cytometric assessment of Annexin V and propidium iodide (PI) staining. Mean + SEM is shown (n = 3–5 mice per group). n.s., not significant. *, p < 0.05 by t-test. (TIF) [file ppat.1006046.s002.tif]

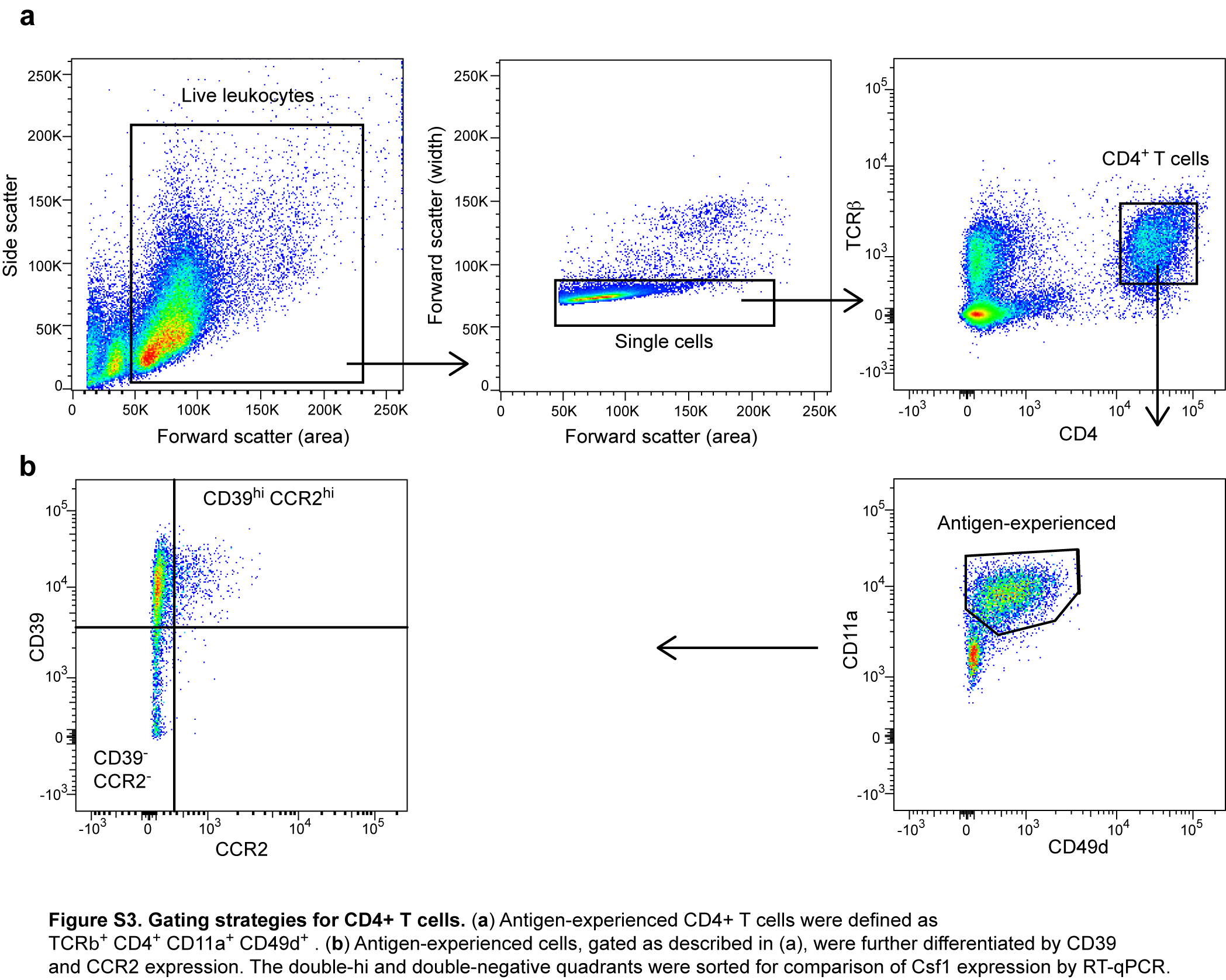

Supplement: S3 Fig — (A) Antigen-experienced CD4+ T cells were defined as TCRβ+ CD4+ CD11a+ CD49d+. (B) Antigen-experienced cells, gated as described in (A), were further differentiated by CD39 and CCR2 expression. The double-hi and double-negative quadrants were sorted for comparison of Csf1 expression by RT-qPCR. (TIF) [file ppat.1006046.s003.tif]

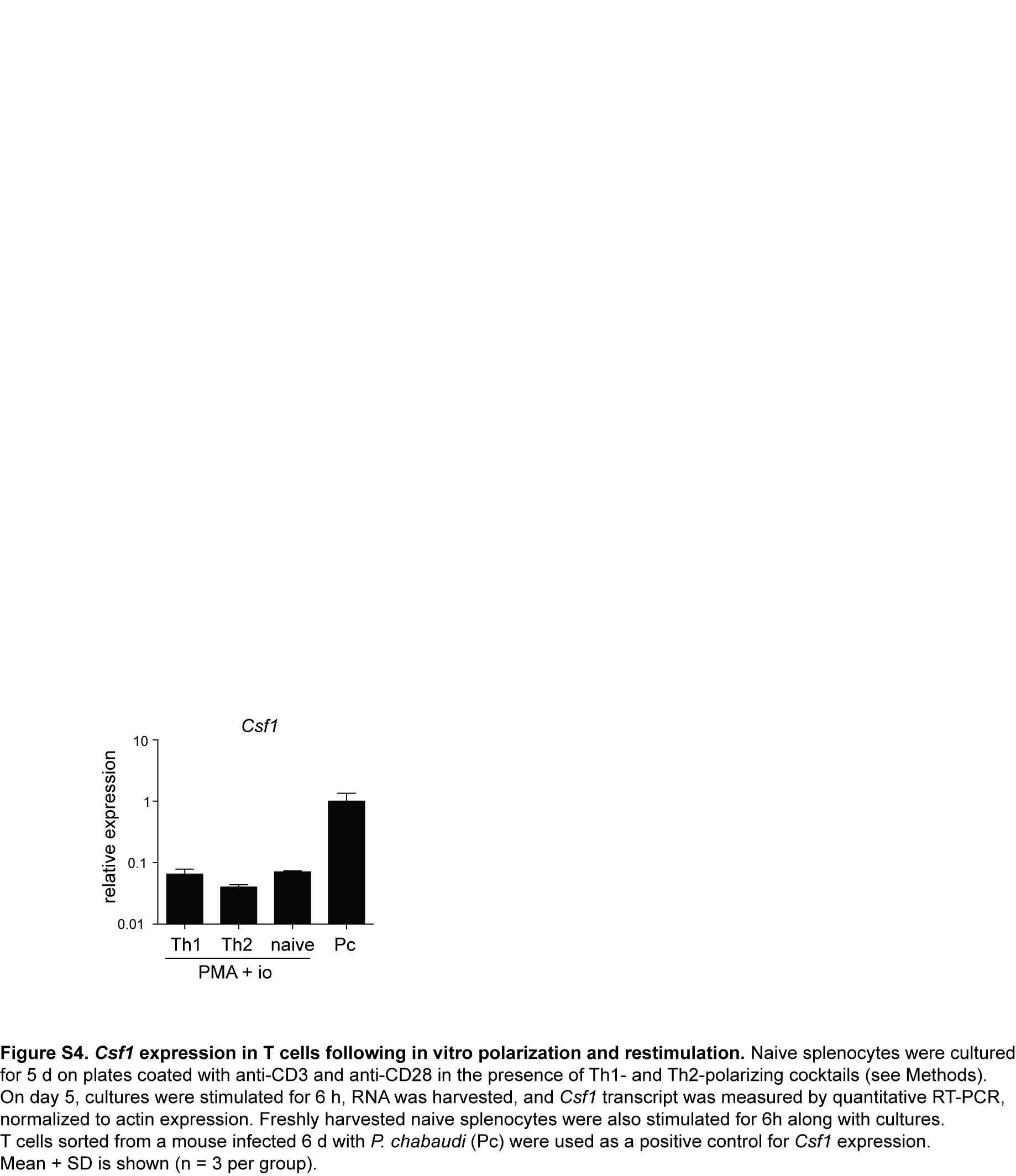

Supplement: S4 Fig — Naïve splenocytes were cultured for 5 d on plates coated with anti-CD3 and anti-CD28 in the presence of Th1 and Th2-polarizing cocktails (see Methods). On day 5, cultures were stimulated for 6 h, RNA was harvested, and Csf1 transcript was measured by RT-qPCR, normalized to actin expression. Freshly harvested naïve splenocytes were also stimulated for 6 h along with cultures. Blood CD4+ antigen-experienced T cells sorted from a mouse infected 6 d with P. chabaudi (Pc) were used as a positive control for Csf1 expression. Mean + SD is shown (n = 3 per group). (TIF) [file ppat.1006046.s004.tif]

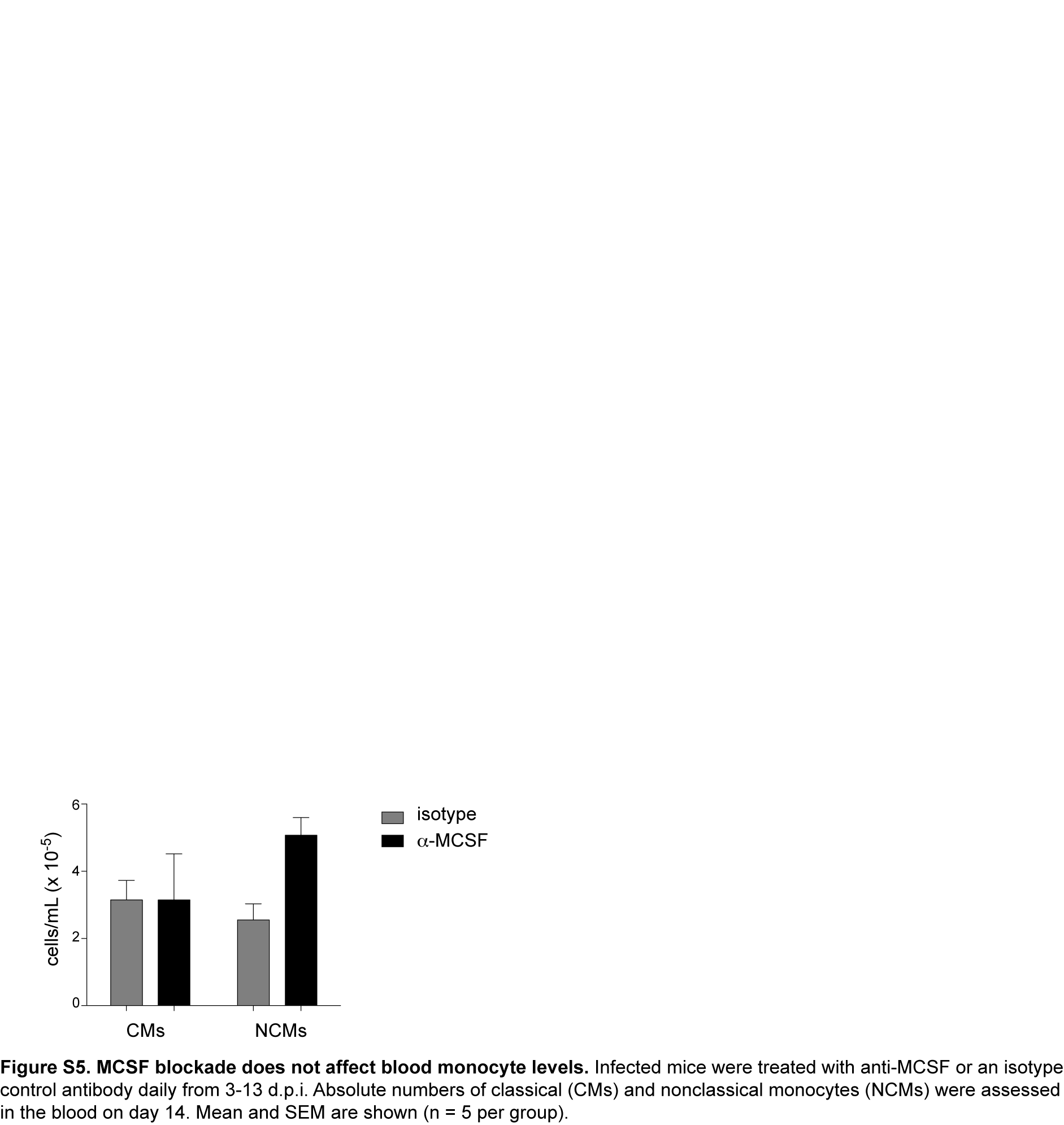

Supplement: S5 Fig — Infected mice were treated with anti-MCSF or an isotype control antibody daily from 3–13 d.p.i. Absolute numbers of classical (CMs) and nonclassical monocytes (NCMs) were assessed in the blood on day 14. Mean and SEM are shown (n = 5 per group). (TIF) [file ppat.1006046.s005.tif]

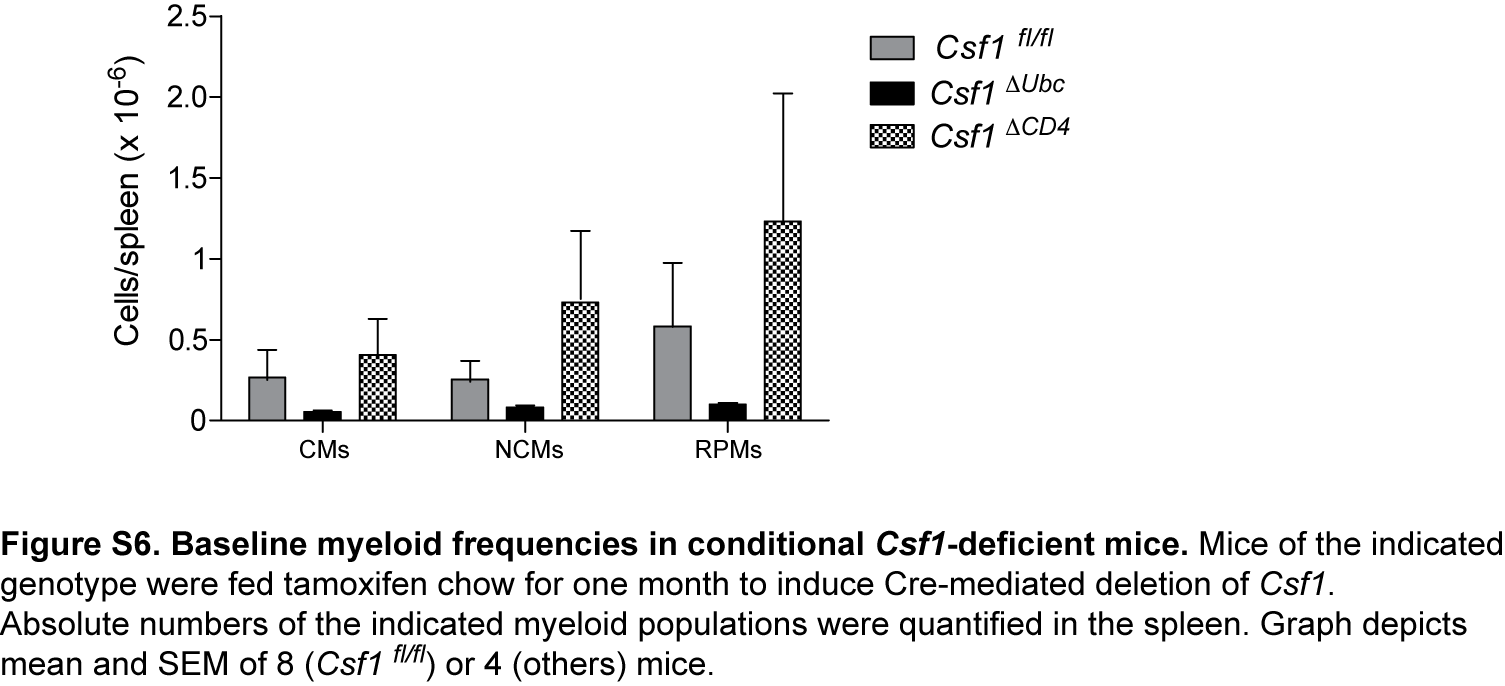

Supplement: S6 Fig — Mice of the indicated genotype were fed tamoxifen chow for one month to induce Cre-mediated deletion of Csf1. Absolute numbers of the indicated myeloid populations were quantified in the spleen. Graph depicts mean and SEM of 8 (Csf fl/fl) or 4 (others) mice. (TIF) [file ppat.1006046.s006.tif]

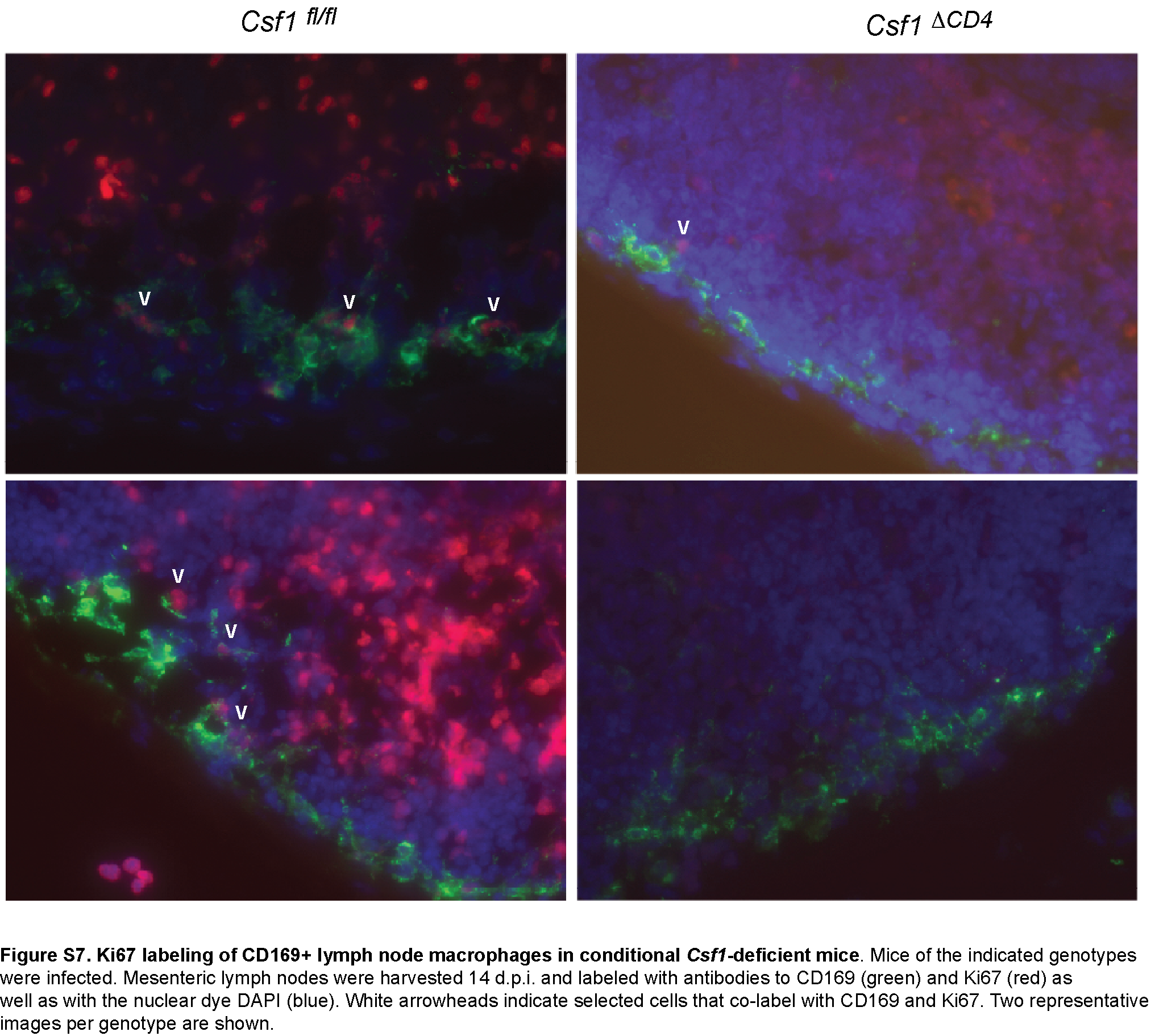

Supplement: S7 Fig — Mice of the indicated genotypes were infected. Mesenteric lymph nodes were harvested 14 d.p.i. and labeled with antibodies to CD169 (green) and Ki67 (red) as well as with the nuclear dye DAPI (blue). White arrowheads indicate selected cells that co-label with CD169 and Ki67. Two representative images per genotype are shown. (TIF) [file ppat.1006046.s007.tif]

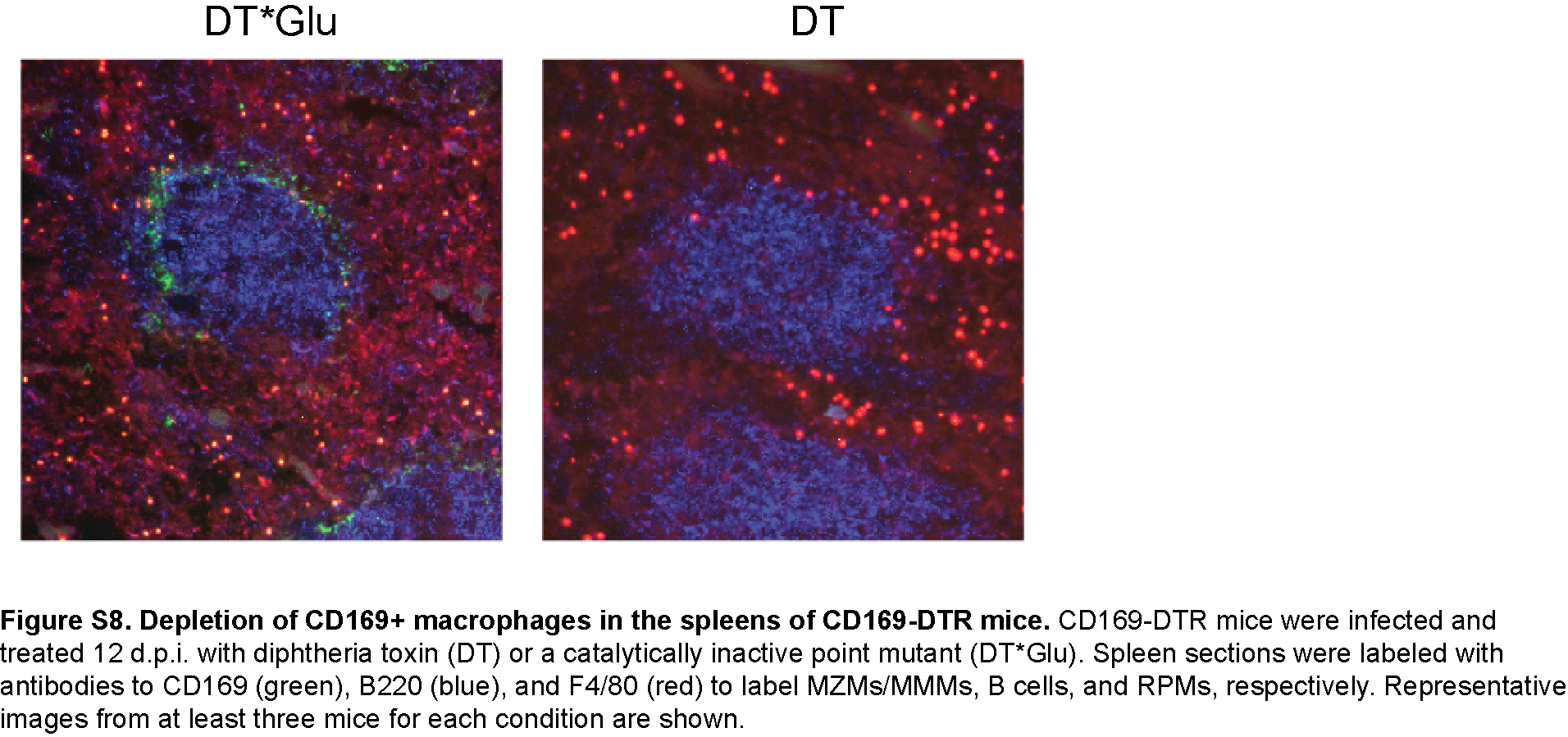

Supplement: S8 Fig — CD169-DTR mice were infected and treated 12 d.p.i. with diphtheria toxin (DT) or a catalytically inactive point mutant (DT*Glu). Spleen sections were labeled with antibodies to CD169 (green), B220 (blue), and F4/80 (red) to label MZMs/MMMs, B cells, and RPMs, respectively. Representative images from at least three mice for each condition are shown. (TIF) [file ppat.1006046.s008.tif]
