# Supplementary material for: Macrophage Colony Stimulating Factor Derived from CD4+ T Cells Contributes to Control of a Blood-Borne Infection
Source: PLoS Pathog. 2016 Dec 6;12(12):e1006046. doi: 10.1371/journal.ppat.1006046 (PMC5140069; doi:10.1371/journal.ppat.1006046)
Supplement: S3 Table — (DOCX) [file ppat.1006046.s011.docx]

| **Antigen** | **Clone** | **Source** |
| --- | --- | --- |
| CD16/32 | 2.4G2 | UCSF Monoclonal Antibody Core |
| CD11b | M1/70 | UCSF Monoclonal Antibody Core |
| F4/80 | BM8 | UCSF Monoclonal Antibody Core |
| LY6c | HK1.4 | eBioscience |
| LY6g | 1A8 | Biolegend |
| Ki67 | SolA15 | eBioscience |
| CD4 | RM4.5 | eBioscience |
| TCRβ | H57-597 | Tonbo |
| CD11a | M17/4 | eBioscience |
| CD49d | R1-2 | eBioscience |
| CCR2 | 475301 | R & D Systems |
| CD39 | 5F2 | eBioscience |
| CD40 | FGK4.5 | UCSF Monoclonal Antibody Core |
| MHCII | NIMR-4 | eBioscience |
| CD68 | FA-11 | ABD Serotec |
| CD115 | AFS98 | eBioscience |

**Table S3. Flow cytometry antibodies used in this study.**
