# Supplementary material for: Macrophage Colony Stimulating Factor Derived from CD4+ T Cells Contributes to Control of a Blood-Borne Infection
Source: PLoS Pathog. 2016 Dec 6;12(12):e1006046. doi: 10.1371/journal.ppat.1006046 (PMC5140069; doi:10.1371/journal.ppat.1006046)
Supplement: S4 Table — (DOCX) [file ppat.1006046.s012.docx]

**Table S4. Quantitative PCR primers used in this study.**

| **Gene** | **Forward Primer** | **Reverse Primer** |
| --- | --- | --- |
| actin | ACCCTAAGGCCAACCGTGAA | CCGCTCGTTGCCAATAGTGA |
| Csf1 (total) | CAGCATGAGGGATCCTCTGA | ACAGAAGAATCCAATGTCTGAG |
| Csf1-variant 1 | TTTTAATTCCATTCCTTTGACTGAC | TTCCATAAAGAGATAGTCCTGTG |
| Csf1-variant 2 | AACAACAGCTTTGCTAAGTGCTCTAG | ACAGAAGAATCCAATGTCTGAG |
| Csf1-variant 3 | AGACATTGGATTCTTCTGTGGG | GAGTACTGTGTCCAGGGCT |
| Ifng | ATGAACGCTACACACTGCATC | CCATCCTTTTGCCAGTTCCTC |
| Il4 | GTC ATC CTG CTC TTC TTT CT | GCT CAC TCT CTG TGG TGT T |

Note that the reverse primers for *Csf1* variant 2 and total *Csf1* are identical. The primer set for variant 2 amplifies all three variants, but generates a 234 bp amplicon from variant 2 versus ~1kb amplicons from variants 1 and 3. Specific amplification is achieved by using a short annealing time; primer specificity was confirmed by gel electrophoresis to check for correct amplicon size.
